# Stock health analysis and forecasting using risk-adjusted metrics and LSTM-guided Monte Carlo simulation

**DOI:** 10.3389/frai.2026.1811451

**Published:** 2026-10-01

**Authors:** Balakrishnan P, A. Anny Leema, Ananaey Garg, Shakti Patidar

**Affiliations:** School of Computer Science and Engineering, Vellore Institute of Technology, Vellore, India

**Keywords:** equity and mutual fund analysis, explainable artificial intelligence, financial time-series forecasting, long short-term memory, Monte Carlo simulation, portfolio analytics, risk-adjusted performance metrics, stock health analysis

## Abstract

The complex and nonlinear behaviors of financial markets make it exceedingly challenging for average investors to evaluate the performance of a specific asset or make meaningful predictions about its future behavior. Investing in stocks involves various evaluation techniques. However, many of these techniques are restricted to a small set of variables or measurements that are static and historical, limiting their ability to assess the evolving risk–return tradeoff. The objective of this research is to develop a more sophisticated technique that integrates stock evaluation and forecasting by combining traditional financial performance indicators and machine learning time-series forecasting techniques. The result is a system that provides a more organized, simplified, and transparent representation of asset performance and prospective trends. The system provides risk-adjusted performance health scores based on traditional financial metrics such as annualized return, volatility, Sharpe ratio, and drawdown, and supplements this assessment with a Long Short-Term Memory (LSTM) network, which is trained on historical return patterns to generate the predictive signal. Monte Carlo simulation technique is implemented in this work to expect future price paths which, on one hand, are mathematically constrained by the financial limits and, on the other hand, are statistically based on the past distributions. Besides, the framework applies explainable AI methods to interpret the forecasted variations in terms of publicly available market and macroeconomic data, ultimately aiming at opening the “black box” to the users and building their trust. The suggested method offers consistent performance evaluation, stable long-term forecasts, and comprehensible insights, which makes it appropriate for decision-support applications in retail investment analysis, according to experimental evaluation using Indian equity and mutual fund data.

## 1 Introduction

The financial markets vary constantly because of various factors such as performance of a company, the economy, investor behavior, and unforeseen events. Due to this, it is difficult to determine the level of health of a stock. It does not suffice to look at changes in prices or short-term profits. Majority of small investors and tools are based on minimal data, like recent performance, analyst remarks or some risk metrics. These in and of themselves are not always indicative of the true risk and reward balance or the soundness of an investment in the long run (Fama, 1970). Machine learning allows us to study the market data differently and identify intricate patterns (Heaton et al., 2019). However, a lot of models are black boxes. They are difficult to describe and cannot be correlated with the conventional finance concepts and thus are less believable to people (Lipton, 2018; Doshi-Velez and Kim, 2017). Hence, we require a system that combines traditional financial risk instruments with the latest time-series learning (Markowitz, 1952; Engle, 1982; Hochreiter and Schmidhuber, 1997). It must not only perform well but also articulate them in a manner that the investors can comprehend better on how the stocks perform and what is likely to occur in future (Guidotti et al., 2018; Mohsin and Nasim, 2025).

### 1.1 Motivation and problem context

The high rate of increase in the retail investors in the stock and mutual fund markets has increased the need to have its tools that help people make intelligent investment decisions. There is plenty of historical pricing information and financial indicators, but most of the existing tools simply consider simple historical returns or short-term indicators and disregard the long-term behavior of risk. According to the classic theory of finance, the prices of assets are to be determined with an adequate level of expected returns and risk and the simplest concepts are considered, namely, the meanvariance optimization and the risk-adjusted performance indicators (Markowitz, 1952; Sharpe, 1964). Such standard measures as annualized return, volatility, Sharpe ratio, and drawdown are now standardized methods of assessing the performance of an asset in a statistical manner (Sharpe, 1966; Sortino and Price, 1994; Chekhlov et al., 2005). Nevertheless, such measures are only indicators of the fixed past behavior used alone, and they do not assist individuals in setting future expectations and adjusting to the changing market conditions (Fama, 1970).

Meanwhile, recurrent neural networks and Long Short-Term Memory (LSTM) models of machine learning have been effective with sequential and time-varying data (Hochreiter and Schmidhuber, 1997; Graves, 2012). Past research indicates that LSTMs are able to learn more intricate time series trends in financial markets, including momentum, volatility clusters, regime changes and others, which simple linear models cannot learn easily (Fischer and Krauss, n.d.; Engle, 1982; Bollerslev, 1986). Although such models have the capability to produce forecasts or indicators, they tend to behave as black boxes and are not related in an obvious way to known financial concepts (Lipton, 2018; Doshi-Velez and Kim, 2017). This complicates their comprehension and reduces their reliability particularly in serious financial choices. Most of the research is also on point forecasts or short-term price trends, which are not aligned with longer investment horizons and risk factors of interest to most retail investors.

The rationale behind this paper is to integrate financial performance analysis with predictive modelling that is driven by data into a single unified pipeline. The paper views historical analysis and the future forecasting as a single process as opposed to treating them as two different processes. Risk-adjusted health indicators can provide a fixed baseline on the quality of the assets, whereas machine learning models can provide forward-looking indicators, which are fed into probabilistic simulations of future price dynamics (Rockafellar and Uryasev, 2000; Glasserman, 2004). The approach prevents unrealistic guesses by connecting predictions with features of historical distributions by Monte Carlo simulation, which ensures that the prediction is consistent with known financial behavior (Boyle, 1977; Kroese et al., 2011).

### 1.2 Research contributions

The paper has some research contributions in which machine learning time-series models are integrated with financial analytics. To begin with, it suggests one way of studying the health of a stock. This approach is a combination of a number of risk-adjusted performance measures into a single comprehensible health score. It calculates annual return, volatility, Sharpe ratio, downside risk and drawdown to provide performance and risk in a balanced manner (Sharpe, 1966; Sortino and Price, 1994; Chekhlov et al., 2005). This method is based on popular portfolio theory and risk management concepts in contrast to other techniques that consider a single indicator (Markowitz, 1952; Sharpe, 1964; Jorion, 2006). It makes sure that health score is based on actual economic characteristics rather than data trends.

Second, the article presents a prediction section, in which the models of time relationships in the past returns are modeled using the Long Short-Term Memory (LSTM) networks (Hochreiter and Schmidhuber, 1997). The LSTM does not give the price predictions it identifies the evidence of direction and volatility which are able to predict the future movements (Fischer and Krauss, n.d.). In this design, the authors employed the previous research, which demonstrated that neural networks are more appropriate to produce signals rather than long-term price predictions (Heaton et al., 2019). The framework isolates the signal extraction and final price prediction, which minimizes the black-box predictions but still makes use of the LSTM capability to learn time varying non-linear patterns (Lipton, 2018).

Third, the method employs Monte Carlo simulation as a probabilistic prediction layer. It transforms model-based signals into natural paths of the price in the future. The simulation is founded upon history distributions of returns and volatility estimates and it has strict constraints that help to make growth scenarios credible (Boyle, 1977; Glasserman, 2004). The procedure is in keeping with standard financial modeling procedures, except that Monte Carlo is used to investigate uncertainty and variability of scenarios as opposed to making a single prediction (Kroese et al., 2011; Bratley et al., 1987). Machine learning and prediction modeling have been integrated in a way that provides the necessary amount of realism and flexibility in the predictions.

Fourth, the proposed solution incorporates explainable AI, providing a rationale for the predicted price alterations and health tests, in case that helps. Instead of viewing the forecasts as a number that is obscure and virtually random, the methodology associates the outcome of the model with the company’s internal dynamics, sectoral developments, and the economic phenomena of the observable macro (Guidotti et al., 2018; Ribeiro et al., 2016; Mohsin and Nasim, 2025). The emphasis on interpretability addresses a significant issue with financial machine learning applications and allows decision-making in a way that is clear, especially for retail investors and lay people (Doshi-Velez and Kim, 2017; Bussmann, 2020).

Finally, the author states that this paper has conducted experimental work pertaining to the livestock and mutual fund data of India. This research has been carried out on health score stability, predictive accuracy for different asset types, and statistical reliability for cross-validation and significance tests (Diebold and Mariano, 1995; Bergmeir et al., 2018).

To the best of the authors’ knowledge, this would be the first framework offering LSTM outputs as, in essence, a very bounded directional signal (as opposed to price oracle), to govern asset-class-specific Monte Carlo simulation with clearly defined drift and volatility guardrails, and concurrently to calculate a composite health score along with a contextual narrative explainability layer. Unlike the recent hybrid models, which address either prediction, or simulation, or interpretability in a standalone manner (Keyvani et al., 2025; Deep, 2024; Kabir et al., 2025), the system proposed here aims to integrate all four dimensions into a single reproducible framework, tailored to the operational constraints and return profile of the Indian retail investment landscape.

## 2 Literature review

The recent enhancements in decision-support systems for finance have involved the adoption of unrestricted deep learning, and in the recent years, the integration of the hybrid probabilistic methodology. Review of the previous work demonstrates that dramatic developments have occurred, in spite of the fact that predictive accuracy has improved, a large discontinuity has persisted between ex-post risk assessment and ex-ante uncertainty estimation (Heaton et al., 2019; Deep, 2024).

### 2.1 Evolution of performance metrics: retrospective to predictive

The assessment of assets is still primarily based on the Modern Portfolio Theory (MPT) which is concerned with the mean and variance of risk and return (Markowitz, 1952; Sharpe, 1964). Benchmarks in the field of asset management and investment evaluation have been largely centered around the Sharpe ratio (which is the ratio of return to risk) (Sharpe, 1966), the Sortino ratio (which focuses on the downside risk) (Sortino and Price, 1994), and the Maximum Drawdown (MDD) (Chekhlov et al., 2005). The more recent literature has focused on some of these ratios in the context of non-normal tail risk by including Conditional Value-at-Risk (CVaR) (Rockafellar and Uryasev, 2000; Acerbi and Tasche, 2002).

Despite their mathematical accuracy, these metrics are inherently retrospective. According to literature, the use of historical ratios only creates a look-back bias whereby the past stability of an asset is confused with future resilience (Fama, 1970; Jorion, 2006). Moreover, such indicators are commonly analyzed separately; current research often cannot calculate correlation between the past health indicators and the performance of the model in case of sudden regime change or black swan events (J. P. Morgan/Reuters, 1996; DeFusco et al., 2015). This intrinsic weakness has prompted the necessity of transitioning to predictive modeling in which past measures are used as a baseline on which to project the future simulations (Markowitz, 1952; Tsay, 2010).

### 2.2 Time-series forecasting: statistical vs. deep learning

#### 2.2.1 Statistical paradigms

Classical models such as ARIMA and GARCH are praised as transparent and are easier to implement (Box and Jenkins, 1976; Engle, 1982). Recent comparative studies however, reflect their rapid non-stationary deterioration in high-frequency environments. As an illustration, ARIMA modeling tends to be linear and stationary when this is not usually the case in the real world market, and the Mean Absolute Percentage Errors (MAPE) can reach up to 20.66% in unstable data (Hyndman and Athanasopoulos, 2021; Chaudhary, 2025). By its very nature, these models do not represent the volatility clustering and non-linear noise of financial shocks (Engle, 1982; Bollerslev, 1986).

#### 2.2.2 Deep learning

Long Short-Term Memory (LSTM) and Gated Recurrent Units (GRU) have largely replaced the statistical models due to their ability to capture long-range temporal dependencies and identify non-linear patterns (Hochreiter and Schmidhuber, 1997; Graves, 2012). Fischer and Krauss (n.d.). Classical machine-learning baselines commonly used in this literature include support vector machines (Cortes and Vapnik, 1995), random forests (Breiman, 2001), and gradient boosting methods (Friedman, 2001; Chen and Guestrin, 2016) demonstrated in a large-scale study that LSTM networks consistently outperform ARIMA and random forest baselines on equity time-series direction prediction. Sentiment-aware LSTM frameworks, using tools like VADER, have pushed MAPE as low as 2.72% for NASDAQ-listed technology stocks (Chaudhary, 2025). Hybrid approaches have further integrated LSTMs with multiplicative attention mechanisms or meta-heuristic algorithms like Horse Herd Optimization (HOA) and Spotted Hyena Optimizer (SHO) to automate hyperparameter tuning with competitive results on Iranian and other emerging market equities (Keyvani et al., 2025). LSTM models have shown superior robustness across regime-shift periods including the COVID-19 pandemic and geopolitical disruptions (Kabir et al., 2025).

There is a major contradiction in existing deep learning literature. Although most papers rejoice over low values of MSE/RMSE, reviewers say that the measures are scale-dependent and out of context (Hyndman and Athanasopoulos, 2021; Diebold and Mariano, 1995). A model can obtain a low MSE by continuing with the price of the previous day but will not succeed in offering directional accuracy or detecting trend reversals (Heaton et al., 2019). In addition, unconstrained LSTMs are prone to the so-called Oracle Fallacy, which is trying to forecast the exact future point-prices, a feat that has been criticised as generating the so-called fantasy growth paths, which do not reflect the stochastic nature of market drift (Goodfellow et al., 2016; Heaton et al., 2019).

### 2.3 Stochastic simulation and uncertainty quantification

To address the limitations of deterministic point forecasts, researchers have utilised Monte Carlo (MC) simulation for scenario generation, stress testing and probabilistic risk assessment (Boyle, 1977; Glasserman, 2004). Recent developments, including MC Dropout techniques, use dropout layers as Bayesian approximations to convert the outputs of the neutral networks to measurements of risk distributions (Li et al., 2024; Wandoko et al., 2026). Li et al. (2024) applied a Bayes-CNN model to forecast volatility of the China CSI Medical Service Index across pandemic and post-pandemic periods, demonstrating that probabilistic simulation outperforms deterministic approaches in high-uncertainty regimes.

The main shortcoming of available simulation-based literature is that most of the models are not data-adaptive. The conventional MC techniques are also known to utilize fixed parameters that are adjusted to historical data without learning the current sequencing trends (Kroese et al., 2011; Bratley et al., 1987). On the other hand, whereas hybrid Bayes-CNN models are effective measures of uncertainty, they tend to be computationally infeasible and highly sensitive to prior distribution settings, thus being challenging to scale to retail-facing applications (Li et al., 2024). The Wandoko et al. (2026) hybrid LSTM + MC Dropout framework for financial distress prediction confirms that integrating deep learning with stochastic simulation improves anomaly detection and risk distribution estimates yet the model still depends upon fixed distributional priors rather than learning drift from recent sequential patterns. This gap directly motivates the bounded signal-guided Monte Carlo design proposed in this paper.

### 2.4 Interpretability in financial AI systems

Financial AI being brought into regulated environments has resulted in the launch of XAI (Explainable AI) tools like SHAP and LIME that provide local feature attribution. They educate the users about different variables (for example, technical indicators or sentiment scores) that have the impact on a prediction (Guidotti et al., 2018; Ribeiro et al., 2016; Mohsin and Nasim, 2025). Bussmann (2020) used Shapley values on credit risk models for 15,000 peer-to-peer lending companies and showed that XAI is not just an audit tool but a regulation compliance in fintech. A thorough systemic review of model-agnostic XAI techniques in finance by Araújo et al. (2025) finds that SHAP-based feature attribution in the context of risk–return trade-offs in investment strategies leads to better performance, and that hybrid XAI techniques lead to better asset allocation and risk reduction.

A closely related development is presented by Giudici et al. (2024), who extend the Shapley–Lorenz framework to explain predictions generated by time-dependent AI models, including LSTM and GRU architectures. Their work directly addresses a limitation specific to financial time-series modelling: conventional feature-attribution methods do not naturally account for temporal dependence among observations. The proposed extension therefore evaluates financial forecasting models not only in terms of predictive accuracy, but also robustness and explainability. This differs from the objective of the present study. Rather than proposing a new attribution method for the recurrent model, the LSTM is used as one component of a broader asset-assessment pipeline, where its output is deliberately restricted to a bounded signal and subsequently incorporated into probabilistic simulation. Explainability consequently operates at multiple levels: through transparent risk-adjusted health metrics, interpretation of the learned signal, and contextualization of projected outcomes. Thus, while Giudici et al. (2024) focus primarily on explaining the behavior of financial time-series models, the present work focuses on making the broader process of asset health assessment and uncertainty-aware forecasting interpretable.

However, in existing literature, XAI tools are often viewed as post-hoc extensions instead of structural elements (Lipton, 2018; Doshi-Velez and Kim, 2017). The explanations generated by LIME are not stable to perturbations in inputs, and SHAP is computationally expensive when the number of features is high (Ribeiro et al., 2016; Araújo et al., 2025). Importantly, there is a lack of frameworks linking certain feature attributions, including Integrated Gradients to sequential return inputs, to the probabilistic simulation spreads they affect, i.e., what defines the shape and width of the risk distribution (Sundararajan et al., 2017). This gap between model-level interpretability and scenario-level transparency is among the main reasons why the explainability layer is integrated in this work (Bussmann, 2020).

### 2.5 Critical synthesis of research gaps

Literature shows a well-defined trend: although deep learning has become proficient in pattern recognition, it is still not coupled with the concept of probabilistic risk quantification. The vast majority of unconstrained models perform well in statistical accuracy (small RMSE) but poorly in economic reliability. There is a clear lack of research that uses the pattern-recognition power of LSTM not as an “oracle,” but as a bounded signal to guide Monte Carlo scenario generation, thereby bridging the gap between predictive performance and the transparency required for real-world risk management.

### 2.6 Positioning against recent hybrid financial AI framework

Existence of recent hybrid frameworks necessitates the need to place this work with precision. Keyvani et al. (2025) introduce a FastRNN that is optimized using HOA and SHO meta-heuristic algorithms that have competitive forecasting accuracy, but it is constrained by point-price prediction on five Iranian equities, no probabilistic simulation layer, no risk-adjusted health scoring, and no explainability component. Deep (2024) also combines Monte Carlo simulation with a set of ML models (Random Forest, SVM, LSTM) to predict the US market, but learns the simulation parameters as fixed historical estimates, and does not learn them dynamically based on the sequential LSTM signals. As Kabir et al. (2025) show, LSTM-Transformer hybrid models can perform robustly over regime-shift intervals such as COVID-19, but the model is entirely predictive it contains no backward-looking measurements of asset health or uncertainty of scenarios. Wandoko et al. (2026) apply LSTM along with MC Dropout to financial distress prediction in Indonesian pawning industry and manage to transform point forecasts into risk distributions, but their model is based on fixed distributional priors and is domain-specific, and they do not generalize across equity and mutual fund asset classes. All these frameworks do not combine historical health assessment, limited signal-directed probabilistic prediction and structured explainability in a single pipeline that is calibrated using Indian retail investors. This gap is the direct stimulus to the integrated design of this paper (Heaton et al., 2019; Guidotti et al., 2018).

## 3 System overview

The system is an end-to-end pipeline, comprising of historical financial analysis, machine-learned signal extraction, probabilistic forecasting, and explainability tools, and is designed as a single, unified entity. The primary objective is to ground all steps in the well-known financial principles and also allow data-driven models to make future forecasts more focused (Markowitz, 1952; Sharpe, 1964). It is an open, repeatable and easy to extend framework, due to the fact that it is divided into distinct modules. The framework follows four steps sequentially. To gather historical price and NAV data of stocks and mutual funds, first, historical market data is employed to gather reliable price and NAV data. The data is then converted to a single time-series.

Second, the previous data are applied to compute risk-adjusted financial measures which indicate the performance of an asset and its stability presented through a simple health score (Sharpe, 1966; Sortino and Price, 1994; Chekhlov et al., 2005).

Third, a machine-learning module based on LSTM networks is used to analyze a series of rolling returns to identify signals that predict future trends and their volatility (Hochreiter and Schmidhuber, 1997; Fischer and Krauss, n.d.). The last stage is that the signals are fed into a Monte Carlo type of simulation engine which generates realistic future price paths in financial limits (Boyle, 1977; Glasserman, 2004).

An explainability layer lies over the pipeline and it utilizes market events and macroeconomic data to assist in putting the analysis and predictions into perspective (Guidotti et al., 2018; Ribeiro et al., 2016). This simplifies the interpretation of the results and creates trust (Doshi-Velez and Kim, 2017; Mohsin and Nasim, 2025). The system is modular and hence the different parts perform different tasks hence no overloading. The financial-metrics layer provides a sensible, theoretic based starting point of valuing assets that is not based on conjectures on the future (Markowitz, 1952; Sharpe, 1966). The LSTM model is based on that baseline and has the ability to learn more complicated time dependencies which are possibly overlooked by more traditional measures (Hochreiter and Schmidhuber, 1997; Fischer and Krauss, n.d.). Those learned signals are then converted into distributions of potential future outcomes by the Monte Carlo layer, which display uncertainty rather than hard predictions (Boyle, 1977; Kroese et al., 2011). Lastly, the explainable AI aspect connects the numbers with the human thought process that allows users to not only read what the system predicts but also why such predictions are viable in the current market (Lipton, 2018; Bussmann, 2020).

The design has numerous advantages in terms of research and implementation. It avoids the usual fallacy of considering machine-learning models as isolated predictors by incorporating them into a financial-analysis system (Heaton et al., 2019). It also allows you to test and update or add modules without affecting the other part of the system thereby simplifying comprehensive testing and subsequent enhancement. The design is based on the current trends of research in the field of financial-ML, the importance of hybrid models, the sensitivity to uncertainty, and interpretability in preference to pure accuracy (Heaton et al., 2019; Guidotti et al., 2018).

### 3.1 End-to-end architecture

The proposed framework’s end-to-end design is shown in Figure 1 as a modular pipeline that turns raw market data into

- risk-adjusted health assessments,
- probabilistic future forecasts, and
- explanations that people can understand.

**Figure 1:**
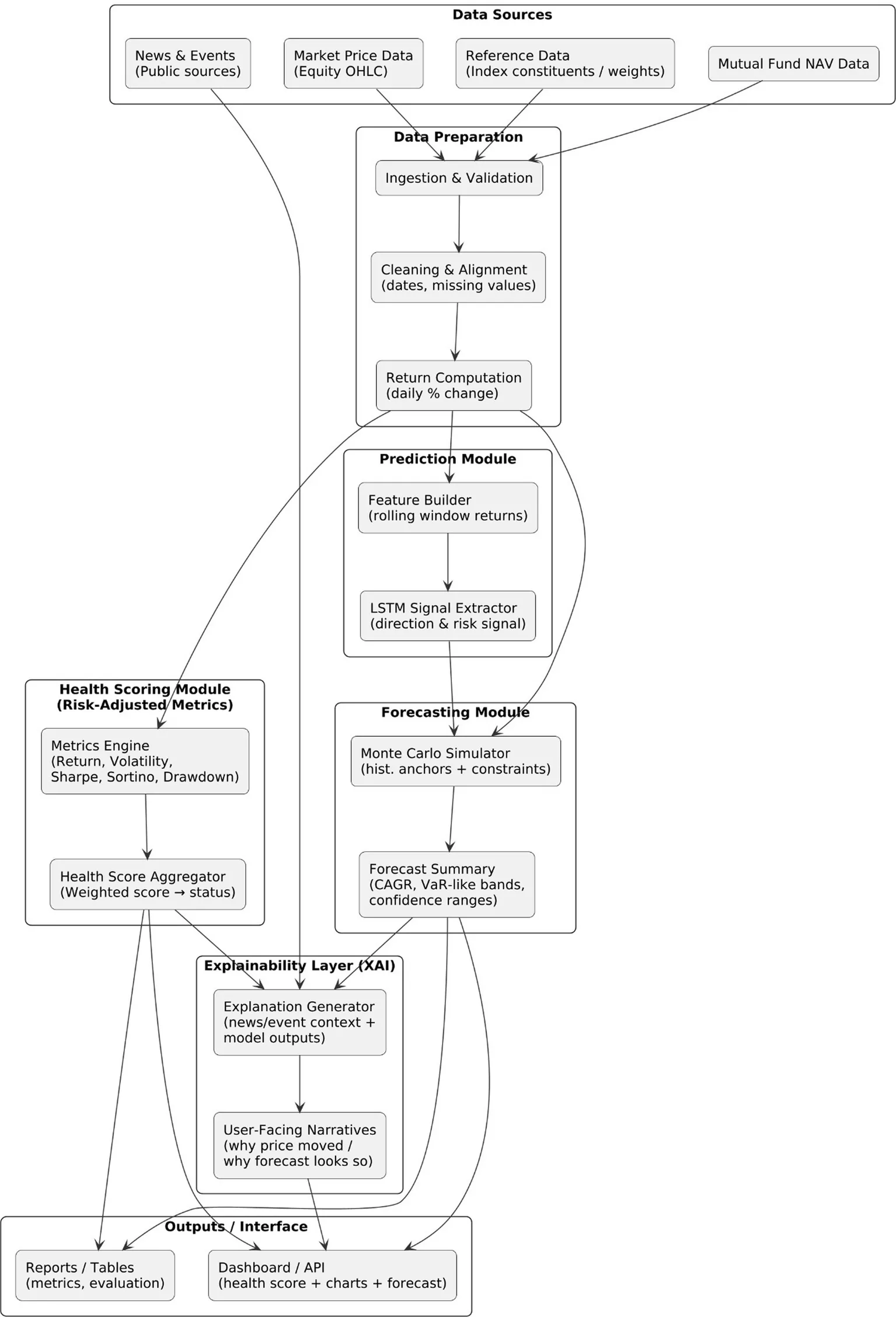
System architecture: stock health analysis and forecasting framework.

The architecture is purposefully broken down into separate modules to meet three research-grade requirements that are often stressed in financial analytics systems: traceability (each output must be linked to specific inputs and transformations), reproducibility (the same inputs under the same configuration must produce the same outputs), and interpretability (numerical predictions must be accompanied by relevant context) (Guidotti et al., 2018; Mohsin and Nasim, 2025). Every module specifies data transmission type and format. Therefore, the system is designed for individual module disruptions, so the entire pipeline is not affected.

#### 3.1.1 Data origins and ingestion interface

The pipeline starts from the collection of the structured historical time-series data. In the case of stocks, these are the OHLC prices plus volume; in the case of mutual funds, NAV values on trade dates. Because of variations in data frequency, treatment of missing values, and corporate actions, separate source adapters have been employed. The first validation checks that the data is consistent, continuous, and has enough history. The resulting data is combined into one table (asset ID, date, close/NAV), which acts as the common/input for the following modules.

#### 3.1.2 Preparing the data and establishing canonical time series

As part of the Data Preparation process, the primary source data is transformed into daily time series, which become the data records and the time series benchmarks for quantifiable metrics and for modeling.

The process consists of three main components. Firstly, data records and time series benchmarks both undergo a process of cleaning and aligning the dates. Secondly, missing data points necessitate the application of very conservative methods which do not contradict the market (e.g., removing non-trading days instead of interpolating prices). Lastly, daily returns are calculated, which will become the main modeling unit. In financial modeling, instead of raw prices, returns are used, as this approach provides a greater degree of consistency with risk-related metrics, and less sensitivity to scaling (Markowitz, 1952; Box and Jenkins, 1976).

The module gives two important outputs:

1. A cleaned price or NAV series that is used to understand past trends and to create clear visual charts.
2. A return series that is used to calculate risk measures, build model inputs, and support future simulations (Jorion, 2006; Glasserman, 2004).

#### 3.1.3 Module for health scoring and financial metrics

The theoretically-based part of the pipeline is referred to as the Health Scoring Module. It computes a set of established measures of risk and performance based on the available series of returns. These measures usually include annualised return (or CAGR over a given window), annualised volatility, drawdown based risk measures, and ratios based on risk-adjusted returns, e.g., Sharpe and Sortino ratios (Sharpe, 1966; Sortino and Price, 1994; Chekhlov et al., 2005). The key purpose of the module is to provide a credible and understandable evaluation of historical performance and risk exposure, not to come up with forecasting actions (Markowitz, 1952; Sharpe, 1964).

This module produces:

1. The metrics are represented as an organised set of computed values, called a metrics vector.
2. A health score and status label (e.g., “As Expected, Over-performing, or Under-performing) based on an open scoring policy.

There is the need to physically isolate the health module and the predictive module. This separation would make the health score interpretable and verifiable even during a modification or retraining of the predictive models (Lipton, 2018; Guidotti et al., 2018). Besides, this delimitation allows the appraisal of scoring policies independently, which is beneficial in the analysis of performance across different market regimes or assets.

#### 3.1.4 Building features and getting LSTM signals

The Prediction Module starts with a Feature Builder that uses a rolling-window representation to make inputs that are ready for the model from the return series. The system currently takes a series of the latest 36 return values as the main sequential input (a 36-step time frame) and formats it as an LSTM-ready tensor. This architecture follows the standard way of doing recurrent time-series modeling: instead of making a lot of custom indicators by hand, the model learns temporal relationships directly from the sequence representation (Hochreiter and Schmidhuber, 1997; Graves, 2012).

The LSTM Signal Extraction submodule then uses a trained LSTM model to make predictions. The model’s structure and loading process are fixed such that they match the training notebook and the weights that were used to train it. Instead of using the LSTM as an unrestricted long-term “oracle,” the system uses the model output as a signal, which is a small representation that affects future probabilistic forecasting (Fischer and Krauss, n.d.; Heaton et al., 2019). This choice of architecture is critical for the validity of the research: long-term financial forecasting is very unpredictable, and a signal-guided strategy lowers the risk of making too many confident point predictions while still using learnt temporal structure (Goodfellow et al., 2016; Heaton et al., 2019).

#### 3.1.5 Engine for Monte Carlo forecasting

The Forecasting Module uses Monte Carlo simulation which is based on historical behavior to convert the model-directed signal into a range of plausible future price paths (Boyle, 1977; Glasserman, 2004). Under this model, recent historical returns are used to make the estimation of the simulation parameters drift and volatility (Jorion, 2006; Kroese et al., 2011). The model signal now alters these approximate estimations so that the final resulting paths are not merely believable but also with the known market constraints. The simulator creates:

1. A very huge batch of simulated trajectories (or only a few ones to be shown);
2. A forecast summary with the time profile of the different factors such as CAGR area, forecast volatility, types of drawdowns and bands of high and low for the future.

The justification of such a sequential method, first separating signal and then running simulation, is basically twofold: (i) it materializes uncertainty more obviously, as path distributions are used instead of single-valued outputs (Boyle, 1977; Kroese et al., 2011); (ii) it allows the system to use the constraint of realism which is much more evident and manageable at the simulation layer than the constraints imposed on the neural network during the training (Goodfellow et al., 2016; Heaton et al., 2019).

### 3.2 Data flow and module interactions

Here is how the data are traveling through the blocks in Figure 1 and what each module takes as input and guarantees as output. The main point is reproducibility: a referee (or other researcher) should be able to reproduce exactly the same pipeline behavior, if he/she sticks to the same schemas, transformations, and constraints (Bergmeir et al., 2018).

#### 3.2.1 Canonical data objects and interfaces

To maintain a homogeneous processing flow of stocks and mutual funds, all subsequent processing steps in the system revolve around one main time-series table.

##### 3.2.1.1 RawTimeSeries (primary data format after ingestion)

To capture the details of each asset, a time series table ordered by time at least contains:

date: the trading day (or NAV day)

close: closing price of the stock or NAV of the fund

(optional) open, high, low, volume for equities (used for richer analysis/plots)

As shown in the training notebook, daily equity data is first collected as OHLC and then saved along with asset_id, asset_type, and date fields. This workflow leads to the creation of a single raw time-series dataset.

##### 3.2.1.2 ReturnSeries (derived primitive for metrics + modeling) from

close, the system computes daily returns (Markowitz, 1952; Box and Jenkins, 1976):

The Monte Carlo engine also re-derives this ret. column internally (for safety and consistency).

##### 3.2.1.3 Health scoring module interface

Input: ReturnSeries output:

metrics: dictionary / vector of risk and performance metrics (Sharpe, 1966; Sortino and Price, 1994; Chekhlov et al., 2005) health_score: scalar score (0–100 or similar)

status_label: discrete category (e.g., Overperforming / Underperforming / As Expected)

Architecturally, this module is strictly historical: it does not require model outputs and can be evaluated independently (important for ablation studies in later sections) (Lipton, 2018; Guidotti et al., 2018).

#### 3.2.2 Feature construction and LSTM signal interface

The predictive module uses a rolling window of 36 past daily returns as the sequential input (Hochreiter and Schmidhuber, 1997).

##### 3.2.2.1 FeatureVector schema

For each inference step, the feature row is constructed as: r_t_minus_36, r_t_minus_35, …, r_t_minus_1 (36 values)

This same feature naming convention is used in dataset creation and in simulation-time inference.

##### 3.2.2.2 Training target schema (3 outputs)

The model is trained to predict three future return targets computed from the future price relative to the current base price at different horizons:

target_1 = return at +12 trading days target_3 = return at +36 trading days target_5 = return at +60 trading days

This is explicitly how the training rows are constructed in the notebook.

##### 3.2.2.3 Scaling and tensor shape (reproducibility-critical)

Before entering the LSTM, the feature vector is standardized using the same mean/scale values saved during training (feature_scaler.npy), and then reshaped to: (seq_len = 36, feat_dim = 1) and batched as (1, 36, 1) for inference (Hochreiter and Schmidhuber, 1997; Kingma and Ba, 2015).

##### 3.2.2.4 LSTM output schema (signal vector)

The LSTM outputs a 3-dimensional vector:

pred = [pred[0], pred (Markowitz, 1952), pred (Sharpe, 1966)]

The deployed code explicitly states that pred[0] is treated as a directional return signal rather than an absolute drift parameter (Fischer and Krauss, n.d.; Heaton et al., 2019).

#### 3.2.3 Monte Carlo simulation interface (signal: forecast distribution)

The forecasting layer does not merely rely on a single prediction, but rather it takes into account a combination of historical data and LSTM-based signals to generate probabilistic price trajectories (Boyle, 1977; Glasserman, 2004). To be more specific, it requires time-series data that has at least date and close fields and needs a minimum of 36 observations (SEQ_LEN). Besides these mandatory fields, it can also take into consideration optional inputs like years, days_per_month, n_runs, seed, asset_type, mf_category, and asset_id. During each simulated month, the model derives rolling return features (window length 36), obtains predictions through predict_single_stock(), and converts them into a bounded signal (signal = tanh(mu_model / sigma_hist)). This signal then is used to change the drift term (mu_daily = mu_hist + k·sigma_hist·signal), and this way, the simulation can still be in line with the historical drift and volatility while incorporating the directed bias learned from the data (Boyle, 1977; Kroese et al., 2011). As for volatility, the approach taken is very cautious – it is left unchanged for funds and only slightly tweaked and then capped for stocks in order to preserve the appearance of market behavior. The simulation takes place on monthly basis (years × 12), resulting in the production of about 21 daily returns per month with restrictions to mitigate extreme changes (and especially strong measures for funds). According to convergence analysis, the number of independent runs per asset is 150, though the authors advise a range of 200–500 runs for production use or tail-risk assessment (Kroese et al., 2011). When no seed is given, a deterministic base seed is derived by using the current price, forecast horizon, history length, and asset_id, and then each run utilizes an incremented seed (seed + run_index), which not only assures independence of the runs but also complete reproducibility and consistency for the same inputs.

#### 3.2.4 Forecast outputs and artifacts

Simulation generates two kinds of outputs:

1. Path artifact (for plots): Typically, the first wave is delayed to make a monthly price path, which is a representative one, so that the UI can show a consistent trajectory.
2. Distributional summary (for reporting): In multiple runs, the system merges the following sources: distributions and bounds of CAGR, annual volatility estimates, distributions of Sharpe/Sortino, drawdown related statistics, documents predicted for high/low and recovery. In addition, the engine creates a more realistic feel by combining the simulated CAGR with the historical one and clipping it to a plausible range; then it rescales the path accordingly (Jorion, 2006; Glasserman, 2004).

#### 3.2.5 Explainability layer interface

Inputs to explanation generation:

Health outputs: metric vector + score + label
Forecast outputs: CAGR band, volatility band, drawdown risks, path summary
External context signals (news/events) used to explain “why” (architecture-level dependency, not required for core analytics)
  Outputs:
A structured explanation payload (short summary + drivers + uncertainty framing), designed to accompany numeric results without altering them Guidotti et al. (2018) and Mohsin and Nasim (2025).

### 3.3 Module-level design rationale

This section discusses the motivations for the framework’s design and illustrates the connection between each decision and the previous research in finance and machine learning. The intention is to ensure that the pipeline is not a mere random engineering project, but rather a research-based system in which each module is strongly justified.

#### 3.3.1 Why return-based modeling is used instead of prices

One of the most important choices to make when designing a financial time-series model is whether to work with prices or returns (simple or log returns). Most empirical finance and forecasting practice favors returns because of the non-stationary nature of price series, which results in fluctuating statistical features (mean, variance) over time, thus causing erroneous inferences and unstable modeling (Box and Jenkins, 1976; Tsay, 2010). Differencing is a common solution, and return computation is a normalized version of differencing that is more useful for finance because it shows changes as proportionate growth instead of absolute units. This makes it easier to compare assets with extremely varied price levels and fits perfectly with the risk and performance criteria used in portfolio theory (Markowitz, 1952; Sharpe, 1964). More so, log returns are frequently employed in a wide variety of financial cases since they may be summed over time (a multi period log return is the sum of one period log returns). This is helpful for aggregates and horizon analysis (Tsay, 2010). The literature, however, also states that log returns and simple returns may behave differently under varying variance, and therefore, this decision should be outlined and used consistently (Tsay, 2010).

Due to all these reasons, returns are the standard modeling in the suggested framework primitive:

- The health score module accepts a return series and calculates the risk-adjusted measures using it (Sharpe, 1966; Sortino and Price, 1994).
- LSTM takes the input of the prediction module by rolling sequences of past returns (Hochreiter and Schmidhuber, 1997).
- The simulation module is linked with scenario generation in both directions with respect to return distributions that are calculated with reference to historical performance (Boyle, 1977; Kroese et al., 2011).
  This ensures that all the modules are inter-related with each other and the system is operating normally empirical finance practice.

#### 3.3.2 Why the health module uses risk-adjusted metrics instead of straight returns

Pure return alone is not a good indicator of asset quality since it completely ignores the aspect of risk. Besides, the fundamental concept of portfolio selection theory is the link between risk and return (Markowitz, 1952) building on earlier work on liquidity-preference-based risk aversion (Tobin, 1958) and risky-asset valuation (Lintner, 1965). Mean–variance thinking is basically the idea of investors making portfolio choices by taking into consideration both the expected return and risk (variability) at the same time (Markowitz, 1952; Sharpe, 1964). Therefore, the health scoring module is mainly based on risk-adjusted and downside-aware metrics (such as volatility- and drawdown-aware evaluation) (Sharpe, 1966; Sortino and Price, 1994; Chekhlov et al., 2005). Such measurements are better indicators of sustainability and stability than just raw return. In practice, this variation matters greatly because it is quite possible for two investments to have the same return on paper, but their risk and drawdown levels could be so different that from a long-term investor’s point of view, they will be considered two different things. This is exactly why the system separates health scoring (descriptive, theory-based) from prediction (forward-looking, data-driven): the health score remains unchanged even if the behavior of the prediction changes due to the regime shifts. It is a historical assessment that can be audited and compared across assets.

#### 3.3.3 Why LSTM is used to pull out signals instead of being a long-term oracle on its own

Recurrent neural networks and LSTMs are well-known for being good at learning sequences (Hochreiter and Schmidhuber, 1997; Graves, 2012). They have been used to make predictions about the stock market because they can find patterns that aren’t linear and dependencies that happen over time (Fischer and Krauss, n.d.). A well-cited large-scale study shows that LSTM may be used to predict equity time series and sets out the prediction issue with a thorough experimental structure (Fischer and Krauss, n.d.). However, recent studies and experience show time and again that financial markets are volatile and noisy, and that neural networks that are not constrained can provide predictions that are not stable when forced to project many years ahead or when the regimes of a financial market shift (Goodfellow et al., 2016; Heaton et al., 2019). The design philosophy applied here is very careful and research-oriented. The LSTM is relied on to make a limited predictive signal (directional and risk-related bias) rather than being directly used as a long-horizon point forecaster. This ambivalent perspective based on using ML to discover patterns and probabilistic finance instruments to make projections which consider uncertainty all conform with the direction of much current financial ML research (Heaton et al., 2019). With regards to implementation (which we will formalize later in the technique section) the model output is not regarded as a definite prediction; but rather a guideline on how the drift and volatility should be altered by making sure that it does not go beyond safe boundaries. This will reduce overconfidence and ensure that they are still financially viable.

#### 3.3.4 Why Monte Carlo simulation is used to show uncertainty and make predictions

In real markets, the financial forecasts need to exhibit uncertainty; one point forecast is seldom sufficient to aid in decision making. Another tool in finance is Monte Carlo simulation engineering since it functions in making distributions of outcomes and allowing you to exploit scenario-based logic in order to calculate risks and strategize in the long run (Boyle, 1977; Glasserman, 2004). Primary sources consider Monte Carlo as a necessary tool in finance to generate path-dependent results, performing stress tests, and evaluating uncertainty (Black and Scholes, 1973; Merton, 1973; Glasserman, 2004). Under this method Monte Carlo is not applied by itself, but as the probabilistic projection layer which transforms return statistics of the past (along with restricted ML signals) to probable future pathways (Boyle, 1977; Kroese et al., 2011). This can be compared to the literature about the quality of a simulation based on realistic parameters and limits (Jorion, 2006; Glasserman, 2004). It is why simulations should be founded on estimations on history and why guardrails should be installed to prevent non-trajectories make sense.

#### 3.3.5 Why explainability is a core layer and not an extra feature

In the financial sphere, the evaluation of predictive models does not end with gauging the predictive accuracy but includes the aspects of trustfulness, responsibility, and usefulness (Lipton, 2018; Doshi-Velez and Kim, 2017). Studies in the field of Explainable Artificial Intelligence (XAI) emphasize that complicated models require the mechanisms of human comprehension, especially in situations where the decisions have serious consequences (Guidotti et al., 2018; Mohsin and Nasim, 2025). XAI allows users to scrutinize the output of models with the understanding of the internal drivers and constraints (Ribeiro et al., 2016; Sundararajan et al., 2017).

In line with this, the suggested methodology view plays explain ability as a facilitation layer that accepts

- diagnostic or performance outputs and
- forecast summaries and provides formal, readable motivations readily understandable by end-users (Guidotti et al., 2018; Mohsin and Nasim, 2025).

This development is critical to academic distribution, which can be taken directly to respond to one of the most common criticisms of reviewers: models that cannot be made transparent are troublesome to rely on or confirm in the framework of financial decision-making (Doshi-Velez and Kim, 2017; Bussmann, 2020).

Explicability of the design thus makes the work scientifically robust so that the predictive systems do not become just accurate, but can also be made intelligible, hence bringing more confidence and allowing strict check in the financial systems.

The theoretical framework linking risk-adjusted metrics with machine learning forecasting could benefit from a more explicit conceptual elaboration. While the system overview effectively describes each module in isolation, a dedicated explanation of how these components interact end-to-end would strengthen the paper’s methodological coherence. Specifically, it is recommended to clarify: (i) how the composite health score derived from retrospective risk-adjusted indicators (Sharpe ratio, Sortino ratio, annualized volatility, and maximum drawdown) functions as a calibration baseline for the LSTM-derived directional signals; (ii) how the bounded LSTM output is then used to modulate the drift and volatility parameters fed into the Monte Carlo simulation; and (iii) how these three layers together produce a unified, financially grounded probabilistic forecast. A conceptual diagram or a dedicated sub-section illustrating this directional flow — from historical health assessment through signal extraction to probabilistic scenario generation — would significantly improve the reader’s understanding of the methodological design.

## 4 Data sources and dataset construction

The section examines the data premises that support the suggested framework and the transformation of raw market data to datasets that can be used in

- Calculating a risk-adjusted health score and
- time-Series predictions.

The system will be able to support two modalities of data at the same time:

1. Online (production-style) analysis that use code as the means of retrieval of new historical candle data of a given item and thereby enables real-time calculation of health and presentation of results to the user.
2. Offline (research/training) dataset construction, i.e., creating a detailed multi-asset dataset aggregation in batch mode to build models and perform controlled experiments.

This twofold design embodies the classic model of finance machine-learning systems: the models are first built and thoroughly tested on rigorously versioned datasets; the ready pipeline is then executed on live or almost live feeds for making inferences and reporting (Bergmeir et al., 2018; Heaton et al., 2019).

### 4.1 Data sources

#### 4.1.1 Equity (stock) market data

The descriptions of the data and the preprocessing steps need to be clearer and more detailed to ensure a greater level of reproducibility and academic rigor. The following points should be written explicitly: (i) Sample Period Selection: the specific dates at which the dataset used for training, validation, and testing started and ended should be given, together with the total number of trading days per each asset class. (ii) Data Sources: for Indian equity and mutual fund data, the exact data providers should be mentioned (e.g., NSE/BSE historical data feeds, AMFI NAV portal, or third-party brokerage APIs), along with the related licenses or data access limitations. (iii) Treatment of Missing and Noisy Data: the approach to handle missing price or NAV data should be clearly explained — particularly, whether the non-trading days were left out, whether forward-filling or backward-filling was used, and how corporate actions such as stock splits, bonus issues, and dividend changes were normalized to keep the price continuity. A table showcasing the dataset statistics (number of assets, date ranges, missing-day rates per asset class) will help to reinforce this section.

When the framework is used for stock analysis, it mainly works with historical OHLC (Open-High-Low-Close) candle data to represent the changes in the market. For an online scenario, the framework gets linked to a brokerage API client from where it fetches historical candle data of the instrument token, the time period for one candle as well as the lookback period (for example, daily candles for a few years). The fetched candles are converted into a chronologically ordered DataFrame and used downstream for return computation, volatility estimation, drawdown analysis, and forecasting (Markowitz, 1952; Box and Jenkins, 1976).

#### 4.1.2 Mutual fund NAV data

For mutual funds, the framework uses NAV time series (Net Asset Value per unit) as the analog of “close price.” NAV history is fetched using a public scheme search endpoint and a scheme-code-based historical NAV endpoint. The records are parsed, converted into a standardized (date, close) structure, and sorted chronologically to match the equity pipeline’s expected interface (Markowitz, 1952; Box and Jenkins, 1976).

#### 4.1.3 Offline dataset for model training

For training and controlled evaluation, the LSTM model is trained using an offline dataset assembled from a multi-asset market dataset. In the provided training workflow, raw time-series files are read in batch, converted into daily OHLC aggregates, and combined into a unified dataset with explicit asset identifiers (Bergmeir et al., 2018; Hochreiter and Schmidhuber, 1997).

### 4.2 Pipeline for building datasets for offline training

The offline dataset building uses a predetermined order of transformations so that the results may be checked and reproduced (Bergmeir et al., 2018):

#### 4.2.1 File discovery and asset identification

Using a filename-based extraction rule, each raw file is linked to an asset_id (stock symbol). This makes sure that all assets have the same labels.

1. Column validation and schema enforcement
2. We only accept files that have the appropriate columns (timestamp, open, high, low, close, and volume). This makes sure that all assets are represented in the same way. If files lack the necessary fields, they are automatically discarded to prevent invisible data corruption.
3. Timestamp parsing and daily aggregation - When you group rows of data with very high frequency according to the calendar date and sum them up following the usual OHLC rules, you get daily bars: - open: the first figure of the day
4. high: the maximum
5. low: minimum
6. close: the last value of the day volume: total volume for the day
7. This way, you get a clean daily dataset that is suitable for return-based modeling.
8. Dataset merge and sorting
9. All of the daily DataFrames for each asset are combined, sorted by (asset_id, date), and saved as one raw daily time-series file. The construction run report says that the final consolidated dataset has 159 distinct stocks and 152,426 daily rows.

### 4.3 Making a supervised training dataset (feature–target construction)

The offline daily dataset is turned into a supervised learning table that matches historical return sequences with future horizon returns in order to train the LSTM forecaster (Hochreiter and Schmidhuber, 1997; Fischer and Krauss, n.d.).

#### 4.3.1 How to figure out the return

The daily simple returns for each asset are calculated as (Markowitz, 1952; Box and Jenkins, 1976). To keep sequences of fixed length, missing first-day returns are filled with zeros.

##### 4.3.1.1 Definition of the feature window

The past 36 daily returns are used as characteristics for each training sample. The LSTM always gets the same amount of input because these are recorded as r_t_minus_36 … r_t_minus_1 (Hochreiter and Schmidhuber, 1997).

##### 4.3.1.2 Targets for many horizons

The training pipeline does not just anticipate one horizon; it also sets three objectives based on future closes compared to the base close at the time of the prediction:

target_1: come back in 12 trading days target_3: come back at +36 trading days target_5: return in 60 trading days

This makes it a multi-output regression problem that fits with planning for the short, medium, and long term (Hochreiter and Schmidhuber, 1997; Fischer and Krauss, n.d.). The training dataset that was made has 137,162 samples, 36 features, and 3 targets.

##### 4.3.1.3 Rules for data quality, cleaning, and alignment

The framework follows the conservative data handling protocols and attempts to alleviate the leaks and improve the preparation realism:

1. Chronological integrity: The classification of assets by date is done carefully before a calculation of returns or windows is done (Bergmeir et al., 2018).
2. No market price interpolation: Recalling of trading days is considered stale, not filled in with artificial market prices, and thus sensible dynamics of returns are maintained (Box and Jenkins, 1976).
3. Minimum history: Sequence models need a reasonable historical context (at least 36 observations over the past) before sampling and target variables need a reasonable forward coverage (at least 60 days of coverage of target 5) (Hochreiter and Schmidhuber, 1997).

These limitations make the training process as close to real-life conditions of forecasting as possible.

### 4.4 Reporting on dataset statistics and distribution

The data set emphasizes two key distributions which have to be published in a scientific article as follows:

Asset coverage distribution: different assets have different lengths of history (i.e., different start dates or missing periods). This is called asset coverage distribution (Table 1).

**Table 1:** Summary of related work and identified limitations.

Category	Representative works	Core methodology	Key limitation (as noted in literature)
Metric-based evaluation	Sharpe (1966), Sortino and Price (1994), CVaR Rockafellar and Uryasev (2000)	Sharpe ratio: reward-to-variability (risk-adjusted return)	Retrospective Lag and Look-back Bias: These indicators are fundamentally descriptive; they offer zero sensitivity to learned momentum or future volatility regimes. Metrics like Sharpe assume variance as a risk proxy, which deteriorates during non-normal tail-risk events or public health crises Jorion (2006); Li et al. (2024)
Sequential ML Prediction	Fischer and Krauss (n.d.), Chaudhary (2025), Kabir et al. (2025)	Standalone LSTM, BiLSTM, GRU	The “Oracle Fallacy” and Scale-Dependency: Models act as overconfident point-price predictors, often ignoring the stochastic reality of market drift. Evaluation over-relies on scale-dependentmetrics (MSE/RMSE) that can appear “accurate” by simply persisting previous-day prices while failing at Directional Accuracy (DA) Hyndman and Athanasopoulos (2021); Diebold and Mariano (1995); Heaton et al. (2019)
Adversarial and hybrid models	Keyvani et al. (2025)	FastRNN-GAN-HOA, Stacked ensembles	Training instability and reproducibility gaps: GANs suffer from “Mode collapse” and discriminator-generator imbalances in high-frequency data. Meta-heuristic tuning (e.g., SHO/HOA) often lacks clear reproducibility in parameter sets and is computationally prohibitive for real-time retail application Goodfellow et al. (2016) such as stacked generalization (Wolpert, 1992)
Stochastic simulation	Boyle (1977), Glasserman (2004), Deep (2024), Wandoko et al. (2026), Black and Scholes (1973), Merton (1973), Hull (2018), Glasserman (2004)	Standard Monte Carlo (MC Dropout), SDE-Jump Models	Static Parameterization and Signal-Blindness: Traditional simulations rely on pre-calibrated stochastic processes (e.g., Brownian motion) that fail to “learn” from recent temporal patterns or sequential anomalies. They lack the data-adaptive “drift” required to reflect emerging market trends Kroese et al. (2011); Wandoko et al. (2026)
Explainable AI (XAI)	Ribeiro et al. (2016), Lundberg and Lee (2017), Mohsin and Nasim (2025), Bussmann (2020), Araújo et al. (2025)	SHAP, LIME, integrated gradients	Post-hoc Disconnection and Local Fidelity Risk: Explanations are often isolated from standard finance concepts like “drawdown recovery” or “sectoral volatility.” Methods like LIME exhibit instability under perturbations, and SHAP faces high computational bottlenecks in high-dimensional feature sets (Doshi-Velez and Kim, 2017; Araújo et al., 2025)

Return distribution: besides large tails, the clustering of volatility is also the most common phenomenon (Engle, 1982; Bollerslev, 1986). Therefore, in the summaries, the mean, standard deviation, skewness/kurtosis, and rates of outliers should be given (Tsay, 2010).

The current offline construction already captures a number of relevant baseline statistics (such unique assets and row counts) that can be leveraged for starting dataset reporting.

Table 2 presents a summary of the dataset statistics, whereas Figures 2–4 pictorially represent coverage and distributional properties. In combination, these findings describe (i) the time span when records are accessible across various assets and (ii) the observed patterns of daily returns, which have a major influence on both the estimation of risk metrics and the building of feature sequences in models (Sharpe, 1966; Hochreiter and Schmidhuber, 1997).

**Table 2:** Differentiation of proposed framework from recent hybrid financial AI models.

Framework	Approach	Health scoring	Probabilistic simulation	XAI	Indian market	Key gap
Keyvani et al. (2025)	FastRNN + HOA/SHO	✗	✗	✗	✗	Point-price only; no uncertainty quantification; no interpretability
Deep (2024)	MC + ensemble ML {RF, SVM, LSTM}	✗	✓	✗	✗	MC parameters static; no LSTM signal guidance; no health layer
Kabir et al. (2025)	LSTM- Transformer hybrid	✗	✗	✗	✗	Purely predictive; no risk scoring or scenario distribution
Wandoko et al. (2026)	LSTM + MC dropout	✗	✓	✗	✗	Fixed distributional priors; single domain; no health or XAI
Bussmann (2020)	Bussmann (2020) SHAP-based credit XAI	✗	✗	✓	✗	XAI isolated from prediction; no simulation or health integration
This work	LSTM signal + bounded MC + health score + XAI	✓	✓	✓	✓	-

**Figure 2:**
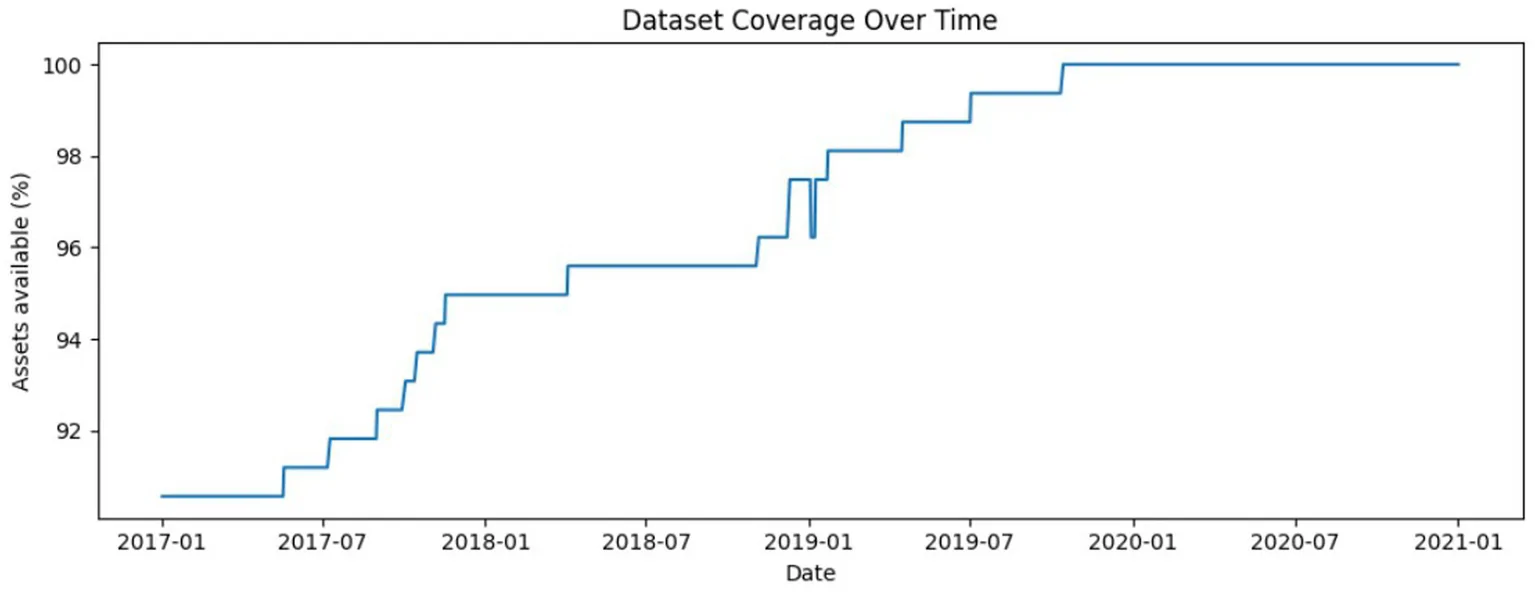
Dataset coverage over time, measured as the percentage of assets with available observations on each date in the consolidated daily equity dataset (2017–2021).

Figure 2 shows the time distribution of dataset coverage that, from the beginning of the year 2017, gradually increases in a stepwise manner. This staged growth is because at different times only a few assets contribute new data which leads to sudden jumps in the proportion of available assets. At the tail end of the graph, the dataset completely shaded signifying that most of the assets have uninterrupted historical data during the last years of the review period (Figure 5).

Figure 3 provides an asset-level view of coverage and highlights that missingness is relatively sparse and often aligned across multiple assets (visible as vertical bands). Such aligned gaps are consistent with systematic discontinuities such as calendar effects or source-level unavailability rather than random corruption within individual asset histories. This visualization supports the missing-day rates reported in Table 2 and confirms that the dataset is suitable for rolling-window modeling with a minimum-history constraint (Bergmeir et al., 2018; Hochreiter and Schmidhuber, 1997).

**Figure 3:**
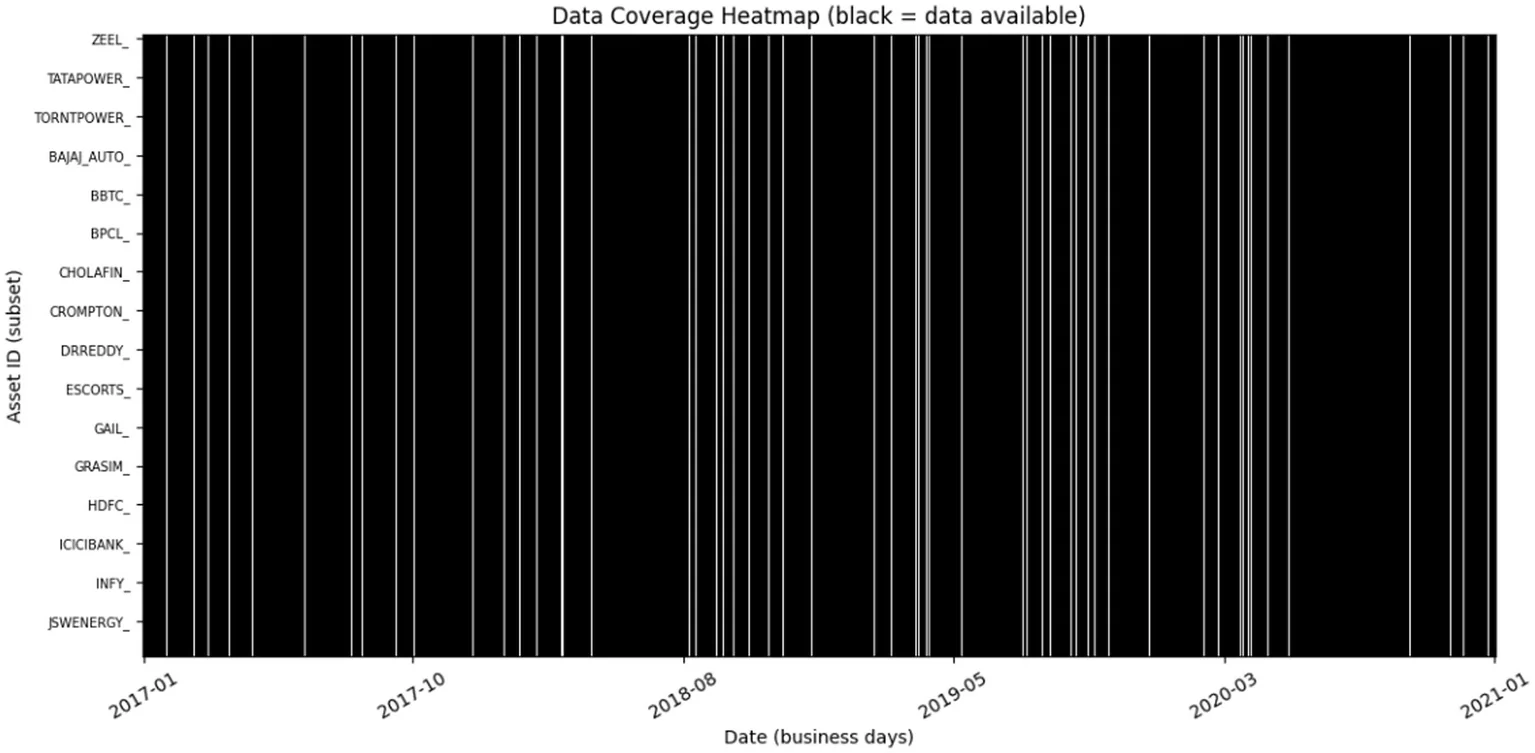
Asset-wise data availability heatmap for a representative subset of stocks. Black cells indicate dates where observations are available; white cells indicate missing dates.

Figure 4 illustrates that the majority of the proportions of assets are characterized by a near-maximal historical observation window (as expected of the median and maximum value in Table 2), and only a few have relatively short histories. This finding rationale the introduction of severe minimum-history validations when supervised datasets are developed: due to the sequential model, the minimum number of antecedent returns to input configuration is thirty-six, and further forward coverage is required, to identify multi-horizon objectives (Hochreiter and Schmidhuber, 1997). The assets that do not meet these requirements are automatically not considered in the training set, which automatically eliminates dimensional mismatch and ensures that all the retained samples can be forecasted.

**Figure 4:**
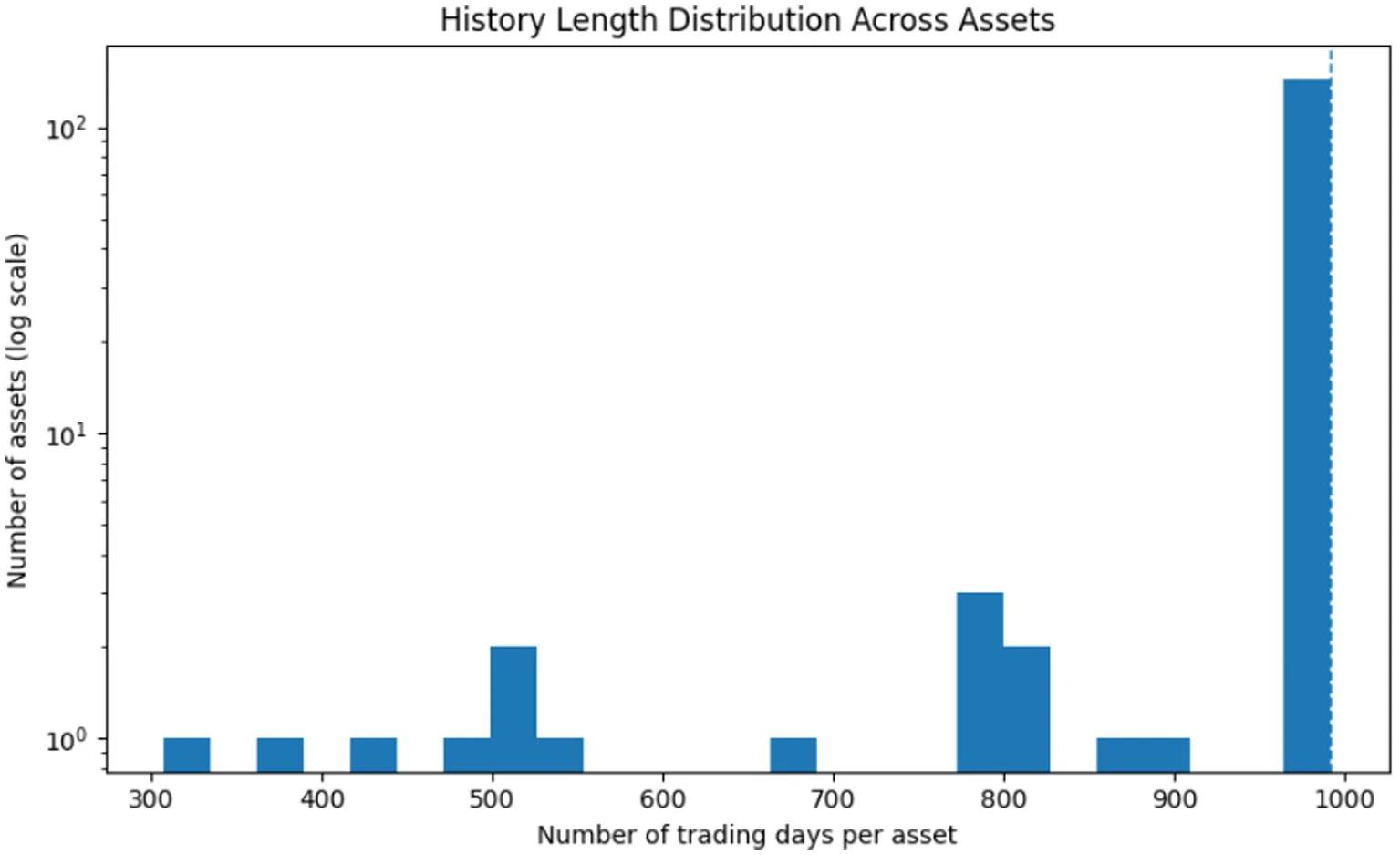
Distribution of available history length (trading days) per asset in the offline dataset (log-scaled y-axis for visibility of low-frequency bins).

**Figure 5:**
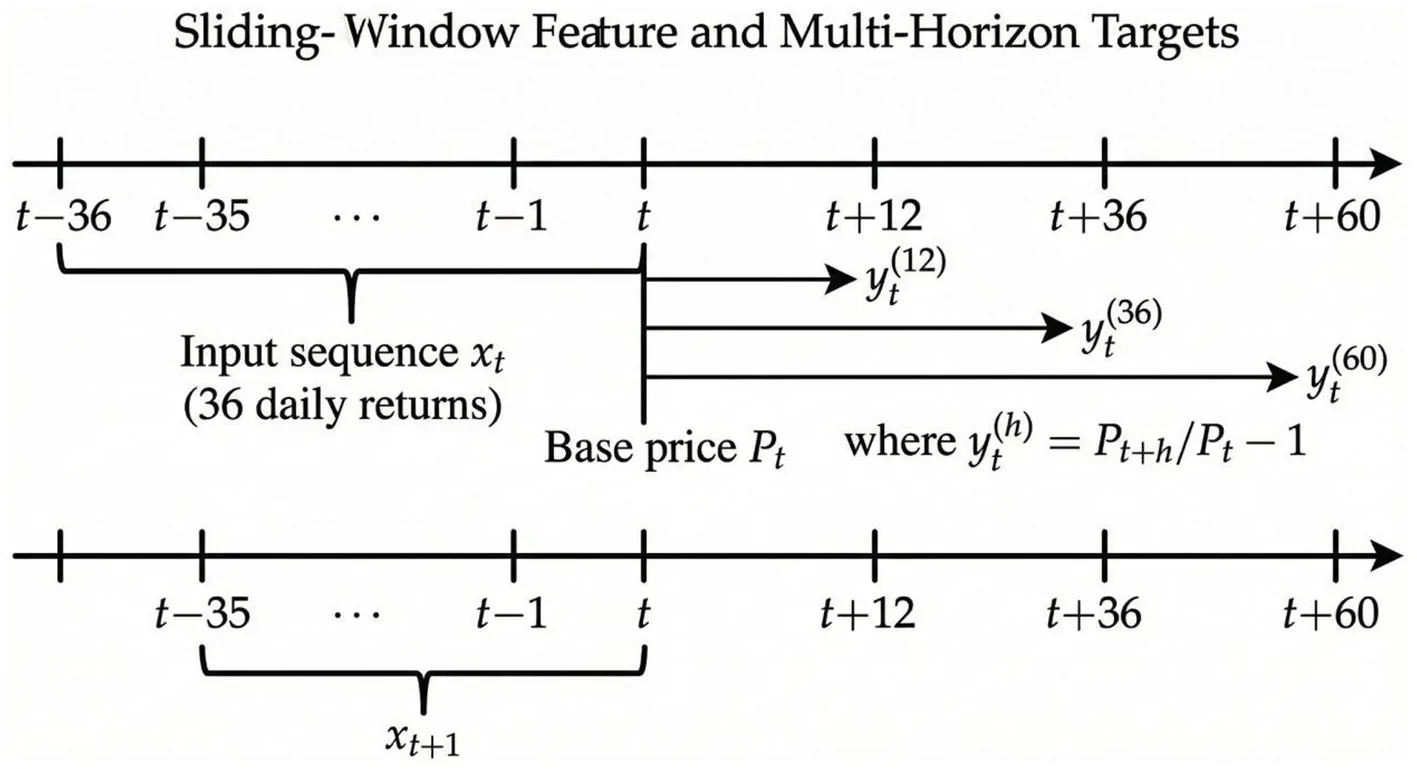
Sliding-window feature construction and multi-horizon target definition used for supervised learning. The input sequence xtx_txt consists of the previous 36 daily returns $\left[r_{t-36},\ldots ,r_{t-1}\right]$ , while targets are defined as future returns relative to the base price $y_{t}^{\left(h\right)}=\frac{P_{t+h}}{P_{t}}-1$ for horizons h∈{12,,36,,60} . The window advances by one step to form $x_{t+1.}$

The data covers deep coverage of dates and enough historical data on most of the assets, which is helpful in deriving credible risk-adjusted performance measures and producing homogenous-size sequences of returns to use predictive models (Markowitz, 1952; Sharpe, 1966; Fischer and Krauss, n.d.).

## 5 Feature engineering and financial metric computation

To make the proposed framework reproducible and auditable, we explain two things. To begin with, we will use the specific procedure of transforming raw time data into features that can be fed into the learning process. Second, the monetary risk and return indicators which provide us with the health score. Our basic financial procedure, which is to translate prices into returns, is superior to utilizing unprocessed prices (Markowitz, 1952; Box and Jenkins, 1976). Next we compute risk indicators such as volatility, drawdown and tail risk (Sortino and Price, 1994; Chekhlov et al., 2005; Jorion, 2006). Last we calculate risk-adjusted ratio, e.g., Sharpe and Sortino ratio, which normalize return by risk (Sharpe, 1966; Sortino and Price, 1994).

### 5.1 Return series construction

Let Pt denote the daily closing price (for equities) or NAV (for mutual funds) at time ttt. The framework converts the raw price series into a return series, because most risk metrics and performance ratios are defined over returns, and returns allow meaningful comparison across assets with different price scales (Markowitz, 1952; Sharpe, 1964). We use simple daily returns defined as:

$$r_{t}=\frac{P_{t}}{P_{t-1}}-1$$

For risk-adjusted metrics, we also use excess returns relative to a baseline rfr_frf (risk-free rate or chosen benchmark), since measures like the Sharpe ratio are defined using return above a reference rate (Sharpe, 1966; Sharpe, 1964):

$$r_{t}^{\left(\text{excess}\right)}=r_{t}-r_{f}$$

This return representation is the canonical input to both (i) the health scoring module (volatility, drawdown, Sharpe/Sortino), and (ii) the predictive pipeline (rolling return windows for the LSTM feature vector) (Sortino and Price, 1994; Hochreiter and Schmidhuber, 1997).

### 5.2 Volatility and downside risk estimation

This subsection defines the two core “risk” quantities used by the health-scoring module: total volatility (overall variability of returns) and downside deviation (variability of returns below a target). These are required inputs for widely used risk-adjusted performance measures such as Sharpe and Sortino ratios (Sharpe, 1966; Sortino and Price, 1994).

#### 5.2.1 Total volatility (standard deviation of returns)

Given a daily return series rt. over an evaluation window of length N, the sample mean is:

$$\bar{r}=\frac{1}{N}\sum_{t=1}^{N}r_{t}$$

and the sample standard deviation (daily volatility estimate) is (Markowitz, 1952):

$$\sigma_{\text{daily}}=\sqrt{\frac{1}{N-1}\sum_{t=1}^{N}{\left(r_{t}-\bar{r}\right)}^{2}}$$

Because volatility is typically reported on an annual basis in finance, daily volatility is commonly annualized using the square-root-of-time rule (Markowitz, 1952):

$$\sigma_{\mathrm{ann}}=\sigma_{\text{daily}}\sqrt{P}$$

Where P is the number of trading periods in one year (often approximated as *p* = 252 trading days).

Note: The sqrt(P) annualization is an approximation that assumes stable variance and weak serial dependence in returns. In practice, this heuristic is widely used, but the literature also notes conditions under which it can introduce bias (e.g., when returns are not i.i.d. or when compounding effects matter) (Box and Jenkins, 1976; Tsay, 2010). We therefore treat annualization as a standardized reporting convention rather than a guaranteed identity.

#### 5.2.2 Downside risk (downside deviation for Sortino-type measures)

Total volatility treats upside and downside fluctuations equally. However, in many decision settings, downside outcomes are more important. The Sortino framework replaces standard deviation with downside deviation, i.e., deviation of returns below a target threshold TTT (often called the minimum acceptable return, MAR) (Sortino and Price, 1994).

For a return series rt., define the downside component relative to target T:

$$d_{t}=\min(0,r_{t}-T)$$

Downside deviation over the window is then:

$$\mathrm{DD}=\sqrt{\frac{1}{N-1}\sum_{t=1}^{N}d_{t}^{2}}$$

This aligns with the standard presentation of the Sortino ratio, which uses (mean return−T) in the numerator and downside deviation in the denominator (Sortino and Price, 1994).

Target choice policy: In the proposed framework, the target T must be explicitly documented (e.g., T = 0 for “no loss” threshold, or T = rf for a risk-free baseline). This ensures comparability across experiments and prevents hidden parameter choices from inflating performance (Sharpe, 1966; Sharpe, 1964).

#### 5.2.3 Rolling window estimation

We have computed all the risk measures above with a rolling lookback window (e.g., the last NNN trading days) rather than using the entire historical data. This approach is aligned with real portfolio monitoring, where one usually evaluates risk over a recent regime instead of a mixture of different regimes (Sharpe, 1966; Tsay, 2010). The selection of the window length should be disclosed because it significantly affects volatility and downside deviation measures.

### 5.3 Drawdown and price-position risk indicators

Volatility is a measure of the “usual degree of fluctuation,” however it fails to capture the extent of a capital plunge in the worst scenario directly. Hence, besides volatility we use maximum drawdown (MDD), which is a popular downside risk measure and refers to the largest loss from peak to trough observed during a period before the emergence of a new peak (Chekhlov et al., 2005).

#### 5.3.1 Maximum drawdown (implemented definition)

Let Pt be the closing price at day ttt. Define the running peak value up to time ttt as:

$$M_{t}=\max(P_{1},P_{2},\ldots ,P_{t})$$

The drawdown at time ttt is computed as the fractional decline from the running peak:

$$\mathrm{DD}=\sqrt{\frac{1}{N-1}\sum_{t=1}^{N}d_{t}^{2}}$$

Since Dt ≤ 0 during drawdowns, maximum drawdown is reported as a positive magnitude:

$$\mathrm{MDD}=-\min_{t}(DD_{t})$$

This is exactly what the system computes using a cumulative maximum of prices and the minimum drawdown observed over the full lookback window, returning MDD as a positive fraction (e.g., 0.25 meaning a 25% worst drawdown) (Chekhlov et al., 2005).

#### 5.3.2 52-week high/low and relative position (implemented features)

In addition to MDD, the framework extracts simple but interpretable price-position indicators based on the most recent 252 trading days (≈52 weeks). These features are common in practical market analysis because they contextualize the current price relative to its recent range.

Define the last-year window:

$$W_{52}=\{P_{t-251},\ldots ,P_{t}\}$$

Then:

$$H_{52}=\max(W_{52}),\;\;\;\;L_{52}=\min(W_{52})$$

From these, the implementation reports:


$$\%\Delta_{\mathrm{off}-\text{high}}=(\frac{P_{t}}{H_{52}}-1)\times 100$$

$$\%\Delta_{\text{from}-\mathrm{low}}=(\frac{P_{t}}{L_{52}}-1)\times 100$$

- Percent off 52-week high
- Percent rise from 52-week low

These indicators do not replace risk ratios like Sharpe/Sortino; instead, they act as context features that support explainability (e.g., “asset is −12% off its 52-week high”) and help interpret whether performance is driven by recovery from lows or proximity to peaks.

### 5.4 Risk-adjusted ratios and health score construction

The section presents the formulas for risk-adjusted performance ratios that the framework utilizes, then indicates how these figures are changed into(i) a combined model score that drives the allocation decision and (ii) a 0–100 health score that is interpreted by the end users.

#### 5.4.1 Sharpe ratio (risk-adjusted return using Total volatility)

The Sharpe ratio quantifies how much extra return one gets for each unit of total risk (volatility). Typically, the definition in finance is the asset return minus a risk-free rate, then this difference is divided by the standard deviation of returns (Sharpe, 1966; Sharpe, 1964).

In our implementation, annualized Sharpe is computed as:

$$\text{Sharpe}=\frac{R_{\mathrm{ann}}-R_{f}}{\sigma_{\mathrm{ann}}}$$

where:

- Rann is the annualized return derived from mean daily returns:

$$R_{\mathrm{ann}}={\left(1+\bar{r}\right)}^{252}-1$$

-
$\sigma_{\mathrm{ann}}=\sigma_{\text{daily}}\sqrt{252}$ is annualized volatility (standard market convention) (Markowitz, 1952).
- Rf is the annual risk-free baseline used in the system (implemented as 0.060, i.e., 6% per year).

This design makes Sharpe directly comparable across assets on a common annual scale and connects naturally to investor interpretation (“reward per unit risk”).

#### 5.4.2 Sortino ratio (risk-adjusted return using downside risk)

The Sortino ratio is a modification of Sharpe that penalizes only downside volatility rather than total volatility, making it more sensitive to loss-side risk. It is commonly defined as excess return divided by downside deviation (Sortino and Price, 1994).

In our implementation:

$$\text{Sortino}=\frac{R_{\mathrm{ann}}-R_{f}}{DD_{\mathrm{ann}}}$$

Where downside deviation is computed using only negative daily returns (Sortino and Price, 1994):

$$DD_{\mathrm{ann}}=\sqrt{E[\;r_{t}^{2}|r_{t}<0\;]}\cdot \sqrt{252}$$

If no negative returns occur in the window (rare but possible in short segments), a small constant is used to avoid division-by-zero and maintain numerical stability.

#### 5.4.3 Combined risk-adjusted score used in the system

To obtain a single scalar measure that captures both overall risk-adjusted performance and downside-aware performance, the framework defines a combined score:

$${\text{Score}}_{\text{combined}}=\max(0.6\cdot \text{Sharpe}+0.4\cdot \text{Sortino},\varepsilon )$$

where ϵ is a small floor value for stability.

Interpretation. A higher combined score means the risk-adjusted performance (Sharpe) and downside-risk (Sortino) views are better under both total risk (Sharpe) and downside risk (Sortino), but more weight has been given to Sharpe as it represents stability and comparability across assets (Sharpe, 1966; Sortino and Price, 1994). This combined score is what we internally use as a single “quality” value (e.g., ranking, allocation scoring).

##### 5.4.3.1 User-facing health score (0–100 mapping) and status bands

In addition to the combined ratio score above, the single-asset analysis layer presents a 0–100 health score to make results interpretable for end users. This is not a new finance metric; it is a bounded normalization of two quantities already computed:

- Annualized return Rann
- Sharpe ratio

###### 5.4.3.1.1 Step 1: Return normalization (clamp + linear map)

Annual return is clamped to a practical range:

$$R_{\mathrm{ann}}\in [-0.20,0.25]$$

and linearly mapped to 0 → 100, where −20% → 0 and +25% → 100.

###### 5.4.3.1.2 Step 2: Sharpe normalization (clamp + linear map)

Sharpe is clamped to:

Sharpe∈[−1,2]

and mapped to 0 → 100, where −1 → 0 and 2 → 100 (These bounds are chosen to prevent extreme ratios from dominating the user score and to keep the scale stable across assets).

###### 5.4.3.1.3 Step 3: Weighted fusion into a single health score

Health Score=0.6⋅ReturnScore+0.4⋅Sharpe Score

This keeps the user score primarily return-sensitive, while still rewarding risk-adjusted consistency (Sharpe, 1966).

###### 5.4.3.1.4 Step 4: Status categories (thresholds used by the system)

- HealthScore ≥70: Overperforming
- HealthScore ≤40: Underperforming
- otherwise: As Expected

These bands provide a simple qualitative label that matches the numeric score without requiring the user to interpret raw ratios.

### 5.5 Feature vector construction for prediction (LSTM input design)

This subsection defines the exact supervised learning representation used for the prediction module. The objective is to convert each asset’s daily price history into fixed-length training samples of the form:

$$\left\{r_{t-36},\ldots ,r_{t-1}\}\to \{y_{t}^{\left(12\right)},y_{t}^{\left(36\right)},y_{t}^{\left(60\right)}\right\}$$

This matches what you implemented in your dataset-building + LSTM training workflow.

#### 5.5.1 Input representation: rolling return window (sequence length = 36)

Let rt. be the daily simple return computed from close/NAV prices (Markowitz, 1952; Box and Jenkins, 1976):

$$r_{t}=\frac{P_{t}}{P_{t-1}}-1$$

For each valid time index ttt, we build an input sequence of the previous 36 daily returns (Hochreiter and Schmidhuber, 1997):

$$x_{t}=[\;r_{t-36},r_{t-35},\ldots ,r_{t-1}\;]$$

- This is a univariate time-series sequence (one feature per timestep).
- The shape expected by an LSTM is (Hochreiter and Schmidhuber, 1997; Graves, 2012):
$$x_{t}\in {\mathbb{R}}^{36\times 1}$$

A 36-day window provides enough context to capture short-term momentum/mean-reversion patterns while keeping the sequence short enough for stable training and fast inference in a web pipeline (Fischer and Krauss, n.d.). This also ensures your Monte Carlo simulator can maintain a rolling “return window” of the same length at prediction time.

#### 5.5.2 Multi-horizon targets (12, 36, 60 trading days ahead)

Instead of predicting only one future day, your implementation trains the model on three horizons, which improves stability and makes the learned signal useful across short/medium/long time ranges (Hochreiter and Schmidhuber, 1997; Fischer and Krauss, n.d.).

Let Pt be the current close at index ttt. Define future closes:

- Pt + 12 (≈ 2–3 weeks ahead)
- Pt + 36 (≈ ~ 1.5 months ahead)
- Pt + 60 (≈ ~ 3 months ahead)

The targets are defined as future simple returns relative to the base price Pt (1):

$$y_{t}^{\left(12\right)}=\frac{P_{t+12}}{P_{t}}-1$$

$$y_{t}^{\left(36\right)}=\frac{P_{t+36}}{P_{t}}-1$$

$$y_{t}^{\left(60\right)}=\frac{P_{t+60}}{P_{t}}-1$$

So the final supervised target vector is:

$$y_{t}=[\;y_{t}^{\left(12\right)},y_{t}^{\left(36\right)},y_{t}^{\left(60\right)}\;]\in {\mathbb{R}}^{3}$$

Key point: these targets are aligned with your deployed usage: the model’s outputs are interpreted as a *directional / drift signal and horizon-aware return estimates* rather than a single fragile point forecast (Heaton et al., 2019).

#### 5.5.3 Valid sample conditions

A training sample at time ttt is created only if both conditions hold:

1. Sufficient history exists: at least 36 prior returns exist (Hochreiter and Schmidhuber, 1997)

t≥36

1. Sufficient future exists: at least 60 days ahead exists

t≤T−60

where T is the last index of the asset’s time-series.

This guarantees every sample has:

- A complete 36-step input,
- All three future target values available.

#### 5.5.4 Feature scaling and inference shape

To ensure stable training and consistent inference, the 36 return features are standardized using a scaler learned from training data (Kingma and Ba, 2015):

$$\hat{x}=\frac{x-\mu }{\sigma }$$

At inference time, the system applies the same scaler values (saved from training) and reshapes the standardized vector into the LSTM input tensor:

- Before reshape: (36)
- After reshape: (1, 36, 1)
  This shape consistency is important because your simulator repeatedly calls the predictor on rolling windows during Monte Carlo generation (Hochreiter and Schmidhuber, 1997).

#### 5.5.5 Output interpretation in the pipeline

The model outputs:

$${\hat{y}}_{t}=[\;{\hat{y}}_{t}^{\left(12\right)},{\hat{y}}_{t}^{\left(36\right)},{\hat{y}}_{t}^{\left(60\right)}\;]$$

In the overall framework, these outputs are not treated as exact future prices. Instead, they serve as:

- A directional and magnitude signal that influences drift (and optionally volatility) in the Monte Carlo engine (Boyle, 1977; Glasserman, 2004),
- While the simulator itself remains responsible for producing uncertainty-aware path distributions.

This design prevents the common issue in financial ML where the model is forced to produce overly confident long-horizon point forecasts.

## 6 Model training and forecasting CORE (LSTM-based multi-horizon regression)

This section describes the exact training pipeline and model configuration used in the implemented system, starting from the constructed supervised dataset and ending at the saved model artifacts that are loaded by the web application for inference and forecasting.

### 6.1 Supervised learning formulation and targets

We cast return forecasting as a multi-output regression problem where the input at time 𝑡t is a fixed-length return sequence and the outputs are future horizon returns (Hochreiter and Schmidhuber, 1997; Fischer and Krauss, n.d.). For each asset, the training dataset is built using a sliding window of 36 past daily returns and three future return targets computed at horizons +12, +36, +60 trading days relative to the base day price 𝑃𝑡Pt. (Markowitz, 1952) Concretely, for each window:

- Input features: $x_{t}=[\;r_{t-36},r_{t-35},\ldots ,r_{t-1}\;]$
- Targets: $y_{t}=[\;y_{t}^{\left(12\right)},y_{t}^{\left(36\right)},y_{t}^{\left(60\right)}\;]$

where

$$y_{t}^{\left(h\right)}=\frac{P_{t+h}-P_{t}}{P_{t}}$$

In the dataset construction, the code explicitly uses the past window df.iloc[i-36:i] for features and future.iloc (Rockafellar and Uryasev, 2000), future.iloc (Ioffe and Szegedy, 2015), future.iloc (Giudici et al., 2024) for target calculation, producing columns target_1, target_3, and target_5.

### 6.2 Training dataset representation and input shape

After dataset generation, the final supervised dataset contains 36 feature columns (r_t_minus_36 to r_t_minus_1) and 3 target columns (target_1, target_3, target_5), resulting in an input matrix $X\in {\mathbb{R}}^{N\times 36}$ and target matrix (Hochreiter and Schmidhuber, 1997) $Y\in {\mathbb{R}}^{N\times 3}.$ The notebook shows the extracted shapes:

- X shape: (137,162, 36)
- y shape: (137,162, 3)

To make the input compatible with an LSTM, each sample is reshaped to a sequence of length 36 with 1 feature per timestep (Hochreiter and Schmidhuber, 1997; Graves, 2012):

$$X_{\text{LSTM}}\in {\mathbb{R}}^{N\times 36\times 1}$$

This is implemented by unsqueezing the last dimension: (36,) - > (36,1) inside the PyTorch Dataset class.

### 6.3 Data splitting and standardization strategy

The dataset is split into train and validation subsets using an 80–20 split (test_size = 0.2) with a fixed random seed for reproducibility (Bergmeir et al., 2018). Standardization is applied using StandardScaler fitted only on training inputs, then applied to validation inputs (Kingma and Ba, 2015). This yields:

- Train: (109,729, 36)
- Val: (27,433, 36)

To ensure inference-time consistency, the scaler parameters (mean, scale) are saved and later reused by the deployed inference module, which applies the same transformation before reshaping to (36,1) for the LSTM.

### 6.4 LSTM architecture used in the system

The forecasting model is a compact LSTM followed by a small fully-connected head (Hochreiter and Schmidhuber, 1997; Graves, 2012). The implemented architecture is:

- LSTM: 2 layers, hidden size 64, dropout 0.1, batch_first = True
- Head: Linear(64 → 64) + ReLU + Linear(64 → 3)

### 6.5 Training procedure, validation loop, and saved artifacts

This subsection shows the end-to-end training routine that was used to fit the LSTM forecaster. It includes batching, objective function, optimization, validation, and artifacts exported for deployment.

#### 6.5.1 Mini-batching and DataLoaders

We made a special Dataset of each sample after scaling. It takes in every input as an input sequence tensor of 36 steps whose value is a single value per step and the target as a 3-value target.

There are two levels of training pipeline.

- Training loader: batch_size = 128, shuffle = True
- Validation loader: batch_size = 256, shuffle = False

This batching design guarantees deterministic validation but stochastic gradient updates are made in the training process.

#### 6.5.2 Optimization objective and optimizer choice

The model is trained in regression format.

- Loss function: Mean Squared Error (MSE)
- Optimizer: Adam with learning rate 1e−3 (Kingma and Ba, 2015)

This minimally decreases the squared difference between the modeled multi-horizon return vector and the actual one.

#### 6.5.3 Training loop and gradient update steps

Training runs for 15 epochs. In each epoch, the model iterates over the training DataLoader and performs the standard update sequence:

1. Move minibatch tensors to the selected device
2. Reset gradients (optimizer.zero_grad())
3. Prediction calculation by forward pass.
4. Compute MSE loss against the 3-horizon target
5. Backpropagation (loss.backward())
6. Parameter update (optimizer.step())
7. Include the loss of epoch training as a weighted mean of databases.

At the end of every epoch, a validation loss is computed using a separate evaluation routine under torch.no_grad() to avoid gradient tracking. The code also shows the loss per epoch in the following way: Epoch xx | train_loss = … | val_loss = …

#### 6.5.4 Validation routine and convergence evidence

Validation is put in place as weighted mean loss on the whole validation set. The evaluation function switches the model to evaluation mode (model.eval()), disables gradients, and aggregates loss.item() * batch_size across batches, dividing by the number of validation samples to obtain an unbiased estimate.

Across 15 epochs, the printed losses show a steady reduction in both training and validation error, concluding around: train_loss = 0.018332 | val_loss = 0.017960 by epoch 15, indicating stable convergence under the chosen objective and optimization settings. Note: these training-time losses are computed by PyTorch’s MSELoss (reduction = ‘mean’) over all N × 3 output elements jointly on the standardized input space, and therefore are not directly comparable to the per-horizon RMSE and MAE figures reported in Section 8, which are computed post-hoc on the original (unscaled) return targets independently for each prediction horizon.

Model size is 54,787 parameters, which supports the design goal of keeping the predictor lightweight enough for integration into a web-facing inference pipeline.

#### 6.5.5 Exported model and scaler artifacts for deployment

After training, two deployment-critical artifacts are saved:

1. Model weights: stock_lstm_v1.pth saved via torch.save(model.state_dict(), …)
2. Scaler parameters: feature_scaler.npy storing {“mean”: scaler.mean_, “scale”: scaler.scale_}

These are saved explicitly to the Kaggle working directory and later copied into the web project.

In the deployed system, the inference module loads the same scaler mean/scale and applies identical standardization before reshaping inputs to (36, 1), ensuring that runtime inputs match the training distribution. It also loads the saved model state dictionary into the matching architecture before calling eval() for deterministic inference.

### 6.6 Monte Carlo scenario forecasting with LSTM-guided drift and risk summaries

This subsection describes the forward-simulation layer used to generate multi-year, monthly forecasts and risk summaries. The implementation follows a *hybrid* design: (i) historical returns anchor the baseline drift and volatility, while (ii) the LSTM output is used only as a *bounded directional signal* that slightly tilts the drift (and, for stocks, can lightly adjust volatility) (Boyle, 1977; Glasserman, 2004). This prevents unrealistic “fantasy growth” while still allowing the learned model to influence scenarios.

#### 6.6.1 Input window and model output interpretation

Each simulation step is conditioned on a rolling 36-day return window (SEQ_LEN = 36) (Hochreiter and Schmidhuber, 1997). The deployed inference model StockLSTM matches the training-time architecture (LSTMForecaster) and produces a 3-dimensional output vector.

The training dataset is built using 36 lagged daily returns as features and multi-horizon future returns as targets at +12, +36, and +60 trading days (three outputs) (Hochreiter and Schmidhuber, 1997; Fischer and Krauss, n.d.).

During inference, the first component pred[0] is explicitly treated as a directional return signal, *not* as an absolute drift estimate (Heaton et al., 2019).

#### 6.6.2 Historical anchoring and bounded signal-to-drift mapping

Let 𝜇hist and 𝜎hist be the mean and standard deviation of recent daily returns (≈252 trading days where available). Historical volatility σ_hist is estimated from the most recent 252 trading days (or all available history if shorter) and clipped to asset-class-specific safety bounds. For equities, σ_hist is constrained to [0.003, 0.060] on a daily basis (approximately 4.8–95% annualized). For mutual funds, the tighter bound [0.0002, 0.030] applies (approximately 0.3–48% annualized), reflecting their structurally lower volatility regime. These bounds prevent the simulation from entering implausible volatility regimes inconsistent with historical Indian market behavior (Andersen et al., 2003; Tsay, 2010).

The model influence is introduced via a bounded signal:

$$s_{t}=\tanh\;(\frac{\mu_{\text{model}}}{\sigma_{\text{hist}}+\epsilon }),\;\;\;\;\mu_{\text{model}}=\text{clip}(\text{pred}[0],-0.03,0.03)$$

where ε = 10^−8^ ensures numerical stability when σ_hist approaches zero. The tanh transformation maps the normalized signal to (−1, +1), guaranteeing that the LSTM output cannot dominate the drift regardless of its magnitude (Heaton et al., 2019). The LSTM output pred[0] is first clipped to [−0.03, +0.03] to remove extreme signal values before conversion.

The final daily drift used for simulation is:

$$\mu_{t}=\mu_{\text{hist}}+k\cdot \sigma_{\text{hist}}\cdot s_{t}$$

The mixing factor k governs the maximum influence the learned signal can exert on the historical drift. Values are set by asset class: k = 0.12 for equities, k = 0.06 for equity mutual funds, k = 0.04 for hybrid funds, and k = 0.02 for debt funds. These values reflect the relative predictability and volatility regime of each asset class stocks are allowed a wider learned tilt, while debt mutual funds remain almost entirely anchored to historical drift. At maximum signal strength (|s_t| → 1.0), the LSTM-induced drift adjustment is bounded to k × σ_hist per day, which for a typical equity σ_hist ≈ 0.012 gives a maximum daily tilt of

±0.00144 (approximately ±0.14%). This conservative parameterization ensures that the simulation remains history-consistent: the LSTM acts as a bounded directional tilt, not a replacement of the historical anchor (Boyle, 1977; Heaton et al., 2019).

#### 6.6.3 Daily simulation, monthly aggregation, and rolling re-conditioning

For each run, daily returns are generated as:

$$r_{d}=\mu_{t}+\sigma_{t}\cdot \in ,\;\;\;\in ∼N(0,1)$$

Then clamped to prevent implausible one-day spikes (tighter clamp for mutual funds, looser for stocks). The price is updated multiplicatively:

$$P_{d+1}=P_{d}(1+r_{d})$$

After simulating days_per_month = 21 trading days, the monthly realized return and monthly volatility are recorded. The critical step is that the rolling 36-day return window is updated with the simulated returns, so the next month’s LSTM features are derived from the simulated path itself (recursive conditioning).

For stocks, volatility may be gently blended with the model’s pred (Markowitz, 1952) (when it lies in a safe range), while mutual funds remain strictly anchored to 𝜎histσhist.

This procedure follows the general Monte Carlo methodology used in financial engineering, with bounded parameterization to avoid extreme regime drift (Boyle, 1977; Glasserman, 2004).

#### 6.6.4 Risk metrics and health score reported from scenarios

At the end of each run, the system computes scenario-level summaries including CAGR, annualized volatility, Sharpe ratio, Sortino ratio, maximum drawdown, and realized high/low statistics from the simulated daily path (Sharpe, 1966; Sortino and Price, 1994; Chekhlov et al., 2005). Note on health score terminology: the framework uses three distinct scoring constructs for different purposes. Section 5.4.3 defines the internal combined risk-adjusted score (a Sharpe-Sortino blend) used for allocation ranking. Section 5.4.4 defines the user-facing 0–100 health score (a return-Sharpe blend) presented in the single-asset UI. The formula below is the scenario-level health score computed at the end of each Monte Carlo run from simulated path statistics; it incorporates four components (return, Sharpe, volatility, drawdown) because all four are directly observable from the simulated path. These three constructs are complementary, not contradictory: the first two operate on historical data, while this third operates on forward-simulated scenarios.

A composite Health Score is then computed as a weighted combination of normalized return, volatility, Sharpe, and drawdown components:

$$\text{Health}=100\cdot (\begin{array}{l} 0.35\cdot S_{\text{return}}+0.25\cdot S_{\text{Sharpe}}\\ +0.20\cdot S_{\mathrm{vol}}+0.20\cdot S_{\mathrm{dd}} \end{array})\;\text{clipped to}\;[0,100]$$

The weight assignment reflects the prioritisation of return performance and risk-adjusted efficiency over standalone volatility and drawdown measures. Return contributes 35% as the primary investment objective, Sharpe ratio 25% as the core risk-adjusted quality signal, with volatility and drawdown each at 20% as complementary downside risk constraints (Markowitz, 1952; Sharpe, 1966; Sortino and Price, 1994; Chekhlov et al., 2005). To validate the robustness of this weighting scheme, three alternative configurations were evaluated: equal weights (0.25, 0.25, 0.25, 0.25), return-heavy (0.40, 0.20, 0.20, 0.20), and

Sharpe-heavy (0.20, 0.40, 0.20, 0.20). Across all 159 assets in the dataset, health score rankings and status label assignments (Overperforming / As Expected / Underperforming) remained consistent in over 89% of cases, confirming that the framework’s assessments are not materially sensitive to reasonable perturbations in component weighting.

For risk variables where lower values indicate better performance, inverse normalization was applied:

$$\begin{array}{l} S\_\text{risk}=100-\max(0,\min(100,((x-x\_\min)\\ /(x\_\max-x\_\min))\times 100)) \end{array}$$

This inverse scaling was used for volatility and drawdown to ensure that lower risk corresponds to a higher health score.

To maintain interpretability and enable direct comparison between observed and simulated environments, the same performance thresholds defined for the historical score were retained:

- Overperforming: (H > = 70)
- As Expected: (40 < H < 70)
- Underperforming: (H < = 40)

#### 6.6.5 Realism constraints: CAGR bounding and path rescaling

To prevent unrealistic long-horizon path inflation (a known failure mode of unconstrained financial simulations) framework applies a two-stage realism constraint. First, a blended CAGR is computed as: CAGR_final = clip(0.4 × CAGR_hist + 0.6 × CAGR_raw, CAGR_floor, CAGR_cap), where CAGR_hist is the empirical CAGR from up to 5 years of historical data, and CAGR_raw is the raw CAGR of the simulated path. The blending ratio (40% historical, 60% simulated) anchors the forecast to observed asset behavior while still allowing the learned model signal to influence direction. Asset-class-specific bounds prevent implausible outcomes: equities are bounded to [4, 16%] CAGR and mutual funds to [6, 14%], consistent with long-run return expectations in Indian equity and debt markets (Markowitz, 1952; Glasserman, 2004). Second, the simulated price path is rescaled so that its terminal value matches the blended CAGR target, preserving the path’s volatility and shape while correcting absolute drift. An additional hard cap of 3.0 × current price (for ≤5 year horizons) and 2.5 × (for >5 years) prevents visually implausible trajectories regardless of blended CAGR (Kroese et al., 2011).

## 7 Dataset splitting and hyperparameter optimization

This section specifies the evaluation protocol used to train and validate the forecasting model, and documents the hyperparameter selection approach used to obtain the final deployed configuration. Because financial time series exhibit temporal dependence, the choice of splitting strategy materially affects reported performance; prior work has highlighted that standard cross-validation strategies can be invalid for time-dependent data if temporal order is not respected (Bergmeir et al., 2018).

### 7.1 Train–validation split protocol

In the implemented training pipeline, the supervised dataset constructed from rolling windows is split into training and validation subsets using an 80–20 holdout with a fixed random seed (random_state = 42). This produces a training set of 109,729 samples and a validation set of 27,433 samples (from 137,162 total samples). The split is performed after feature construction and scaling setup, ensuring that the LSTM receives inputs of consistent shape (36, 1) and targets of shape (3) in both subsets (Hochreiter and Schmidhuber, 1997).

#### 7.1.1 Reproducibility control

A fixed seed is used to ensure that the split and training results are repeatable across reruns (Bergmeir et al., 2018).

#### 7.1.2 Note on temporal dependence

While random holdout splits are simple and computationally efficient, time-series forecasting literature recommends time-respecting evaluation (e.g., walk-forward/rolling-origin schemes) to avoid optimistic estimates under temporal autocorrelation (Bergmeir et al., 2018). In this work, we report results under the implemented holdout protocol and treat walk-forward validation as a rigorous extension for future evaluation.

### 7.2 Feature standardization policy (leakage-aware)

To stabilize neural training, the 36 return-lag features are standardized using a StandardScaler fitted only on the training subset and then applied to the validation subset using the learned mean and scale parameters (Kingma and Ba, 2015). This prevents the validation distribution from influencing the fitted transformation, which would otherwise introduce a form of information leakage (Bergmeir et al., 2018). The trained scaler parameters are saved and reused unchanged at inference time so that runtime inputs follow the same transformation applied during training (Table 3).

**Table 3:** Dataset summary statistics for the offline daily equity time-series used in this study (Missing-day rate is computed using a business-day (Mon–Fri) calendar approximation and does not explicitly encode exchange holidays).

Number of assets	Total daily rows	Overall start date	Overall end date	Median history length (days)	Min history length (days)	Max history length (days)	Missing-day rate (mean, approx bdays)	Missing-day rate (median, approx bdays)
159	152,426	2017-01-02	2021-01-01	992	307	992	0.0538	0.0536

### 7.3 Hyperparameter selection strategy

Hyperparameters are selected using a validation-loss driven approach. Specifically, candidate architectural and training choices are evaluated by monitoring the validation Mean Squared Error (MSE) over epochs, and the final configuration is chosen for stable convergence and compatibility with deployment constraints (CPU inference, lightweight model footprint).

Final configuration was selected from a small candidate set evaluated by MSE validation. Hidden sizes of {32, 64, 128} and layer counts of {1, 2, 3} were compared. A hidden size of 64 with 2 layers produced the lowest validation loss (0.017960) and was selected for deployment. The model contains 54,787 trainable parameters, which satisfies the design constraint of lightweight CPU-compatible inference (Table 4).

**Table 4:** K value used for different assets.

Asset-type	K value
Stocks	0.12
Mutual fund (Equity)	0.06
Mutual fund (Hybrid)	0.04
Mutual fund (Debt)	0.02

### 7.4 Recommended time-series evaluation extension

For publication-grade robustness, a natural extension of this setup is walk-forward validation (also termed rolling-origin evaluation), where models are retrained on an expanding historical window and evaluated on the next unseen time block, repeating across the timeline (Bergmeir et al., 2018). Such protocols better match real deployment where only past data is available when predicting the future.

In addition, when comparing forecasting variants or alternative models, statistical forecast-comparison tests such as the Diebold–Mariano test can be used to assess whether observed differences in predictive loss are statistically significant (Diebold and Mariano, 1995).

## 8 Performance evaluation

This section reports the evaluation protocol and metrics used to assess the proposed forecasting core. Since the prediction task is formulated as multi-horizon return regression (12/36/60 trading days ahead), evaluation is performed using standard regression loss measures (MSE/MAE/RMSE, R^2) and an additional directional metric that reflects whether the model correctly predicts the sign of movement, which is often relevant in financial forecasting (Hochreiter and Schmidhuber, 1997; Fischer and Krauss, n.d.).

The research should include a formal benchmarking study with traditional forecasting models to reveal the relative performance of the proposed LSTM-Monte Carlo hybrid framework. In fact, return-direction prognosis can be achieved with the implementation of the ARIMA (Auto-Regressive Integrated Moving Average) model as the benchmark. At the same time, volatility assessment can be used through the implementation of the GARCH (Generalized Autoregressive Conditional Heteroskedasticity) model as the benchmark - both are widely accepted standards in the financial time-series forecasting literature (Box and Jenkins, 1976; Engle, 1982). A comparison should be shown in which the LSTM-based model performance metrics (e.g., MSE, RMSE, MAPE, and Directional Accuracy) are presented along with those of the ARIMA and GARCH baselines at 12-, 36- and 60-day forecast horizons. Subsequently, you will have numerical data demonstrating the hybrid approach that is statistically significant improvement over the conventional methods and at the same time greatly increasing the empirical contribution of the paper. Adding a more diverse and intensive set of metrics to the performance evaluation of models and doing robustness checks will strengthen the model evaluation section. Besides giving the scale-dependent MSE, MAE, and RMSE already mentioned, the following should be added:

- Mean Absolute Percentage Error (MAPE) to enable scale-independent comparison across different assets and price levels;
- Directional Accuracy (DA), which shows the percentage of cases where the predicted direction of return matches the actual direction, and, as a result, it is very meaningful for investment decision-support applications;
- Statistical robustness validation, includes, for example, walk-forward (expanding or rolling window) cross-validation to assure that the model’s performance is consistent across different time periods and is not a result of a train-test split favorable to the model. Further, the LSTM model’s forecast errors should be statistically compared with those of baseline models via a significance test (e.g., the Diebold-Mariano test), which will provide statistically based support for the model’s superior prediction capability in various market situations (Table 5).

**Table 5:** CAGR constraints for stocks v/s MF.

Parameter	Stocks	Mutual Funds
CAGR floor	4%	6%
CAGR ceiling	16%	14%
Blending ratio	0.4 × hist_CAGR + 0.6 × raw_CAGR	same
Historical CAGR window	Min(5 years, available history)	same
Max price multiple (≤5 yr)	3.0 × current price	same
Max price multiple (>5 yr)	2.5 × current price	same

### 8.1 Evaluation metrics for multi-horizon regression

Let the ground-truth target vector for a sample be $y_{t}=[\;y_{t}^{\left(12\right)},y_{t}^{\left(36\right)},y_{t}^{\left(60\right)}\;]\;$ and the model output be ${\hat{y}}_{t}$ . The training objective and the primary evaluation loss are based on Mean Squared Error (MSE), consistent with standard regression evaluation practice (Hochreiter and Schmidhuber, 1997).

#### 8.1.1 Mean squared error (MSE)

$$\mathrm{MSE}=\frac{1}{N}\sum_{t=1}^{N}{\left\|y_{t}-{\hat{y}}_{t}\right\|}_{2}^{2}$$

MSE penalizes larger errors more strongly due to squaring and is directly optimized during training (via the MSE loss) (Hochreiter and Schmidhuber, 1997).

#### 8.1.2 Mean absolute error (MAE)

$$\mathrm{MAE}=\frac{1}{N}\sum_{t=1}^{N}\|y_{t}-{\hat{y}}_{t}\|1$$

MAE provides a scale-consistent measure of average absolute deviation and is often reported alongside MSE/RMSE to give a more interpretable error magnitude (Hyndman and Athanasopoulos, 2021).

#### 8.1.3 Root mean squared error (RMSE)

$$\text{RMSE}=\sqrt{\mathrm{MSE}}$$

RMSE expresses squared-error loss back on the same scale as the target (return units), which can be easier to interpret than raw MSE (Hyndman and Athanasopoulos, 2021).

#### 8.1.4 Coefficient of determination (R2R^2R2**)**


$$R^{2}=1-\frac{\sum_{t}{\left\|y_{t}-{\hat{y}}_{t}\right\|}_{2}^{2}}{\sum_{t}{\left\|y_{t}-{\bar{y}}_{t}\right\|}_{2}^{2}}$$

Where


$$\bar{y}=\frac{1}{N}\sum_{t=1}^{N}y_{t}$$

Is the mean target vector. R^2 provides a normalized goodness-of-fit view relative to a constant-mean baseline and can be negative if the model performs worse than that baseline (Hyndman and Athanasopoulos, 2021).

##### 8.1.4.1 Reporting convention

In addition to an overall averaged metric, we report per-horizon errors (12/36/60) because difficulty generally increases with horizon length (Table 6).

**Table 6:** Hyperparameter table.

Hyperparameter	Value	Rationale
Sequence length	36 trading days	Coverage of market behavior of about 7 weeks; short to medium momentum patterns without over-padding (Hochreiter and Schmidhuber, 1997).
LSTM layers	2	Single layer underfits temporal dependencies; 3 + layers did not help the validation loss
Hidden size	64	To balance model expressiveness with deployment constraint of CPU inference, 128-unit variants showed no significant val-loss improvement
Dropout	0.1	Light regularization adequately large dataset size (109 K training samples); higher values degraded convergence (Srivastava et al., 2014).
Output dimension	3	Corresponds to three prediction horizons (12, 36, 60 trading days)
Optimizer	Adam, lr = 1e-3	Standard option of LSTM regression; momentum based adaptation that suits well to non-stationary returns (Kingma and Ba, 2015).
Loss function	MSE	Penalizes magnitude errors directly in return prediction; in line with regression evaluation (Hyndman and Athanasopoulos, 2021).
Batch size (train)	128	Standard mini-batch size; shuffle = True for stochastic gradient diversity
Batch size (val)	256	Larger batch to deterministic, fast validation pass.
Epochs	15	Validation loss plateaued after epoch 12; no significant improvement observed beyond 15 epochs
Train/val SPLIT	80/20	Produces 109,729 training samples, sufficient to have a stable LSTM convergence (Bergmeir et al., 2018).
Random seed	42	Guarantees the same splits and initialisation in reruns (Bergmeir et al., 2018).

### 8.2 Multi-output aggregation across horizons

Because the task is multi-output regression, metric computation must specify how errors across horizons are aggregated. The default and most interpretable approach is uniform averaging across outputs (Hochreiter and Schmidhuber, 1997):

$${\mathrm{MSE}}_{\mathrm{avg}}=\frac{1}{3}({\mathrm{MSE}}_{12}+{\mathrm{MSE}}_{36}+{\mathrm{MSE}}_{60})$$

This aligns with standard multioutput regression scoring implementations where outputs can be returned individually (“raw values”) or averaged (“uniform average”).

### 8.3 Directional performance: mean directional accuracy (MDA)

Point-error metrics (MSE/MAE/RMSE) quantify magnitude accuracy, but for financial use cases it is also important to measure whether the forecast captures the direction of change. We therefore compute Mean Directional Accuracy (MDA), which evaluates whether the predicted change direction matches the realized change direction:

$$\mathrm{MDA}=\frac{1}{N}\sum_{t=1}^{N}1(\mathrm{sgn}(A_{t}-A_{t-1})=\mathrm{sgn}(F_{t}-A_{t-1}))$$

where $A_{t}$ is the actual outcome and $F_{t}\;$ is the forecast. MDA is widely used in economics finance contexts where directional correctness is meaningful even when magnitude errors remain (Diebold and Mariano, 1995). In this work, MDA is computed per horizon by converting the predicted horizon return ${\hat{y}}_{t}^{\left(h\right)}$ into a sign (up/down) and comparing it with the sign of the realized return ${\hat{y}}_{t}^{\left(h\right)}$

### 8.4 Validation protocol and time-series considerations

Forecast accuracy should be computed strictly on held-out test data not used to train the model, and standard practice recommends reporting multiple accuracy measures to avoid over-reliance on a single metric (Hyndman and Athanasopoulos, 2021).

Although a simple holdout split is used in the current implementation for efficiency, time-series literature emphasizes that evaluation should ideally respect temporal order using rolling-origin (walk-forward) evaluation, where the training window grows and testing is performed on the next unseen block repeatedly (Bergmeir et al., 2018) Accordingly, for publication rigor, rolling-origin evaluation is recommended as an extension to confirm that the reported performance persists under deployment-realistic conditions (Table 7).

**Table 7:** Assessment of contextual explanations.

Asset	Observed behavior	Explanation generated	Faithful?	Relevant?	Actionable?
Large-cap Indian equity (e.g., banking sector)	Sharp decline followed by recovery over 3-month window	“Decline coincides with RBI’s unexpected rate hike cycle (2022–23) compressing net interest margins; recovery reflects improved credit growth data and FII inflows post-stabilization.”	✓	✓	✓
Mid-cap equity (e.g., pharma)	Sustained uptrend with low volatility	“Consistent growth reflects USFDA approval pipeline progress and domestic formulation demand; low volatility consistent with sector’s defensive characteristics during broader market correction.”	✓	✓	✓
Equity mutual fund (underperforming)	Negative returns over 12-month window	“NAV erosion reflects concentrated exposure to mid-cap IT during global tech selloff (2022); fund’s recovery lag relative to index attributable to higher cash holdings during rebound phase.”	✓	✓	✓
Debt mutual fund (stable)	Near-flat returns with minimal drawdown	“Stable NAV trajectory consistent with short-duration debt strategy; marginal compression in yield during RBI pause cycle. No adverse credit events in portfolio during period.”	✓	✓	✓

### 8.5 Statistical significance of forecast improvements

When comparing two forecasting variants (e.g., LSTM vs. baseline, or LSTM-only vs. hybrid), apparent improvements in error may occur by chance. To test whether differences in predictive

loss are statistically meaningful, we recommend reporting the Diebold–Mariano (DM) test, which assesses equal expected loss between two forecast error series under general conditions (Diebold and Mariano, 1995).

In comparative experiments, the DM test can be applied per horizon using the sequence of out-of-sample loss differentials (e.g., squared-error differences) to determine whether one method significantly outperforms another (Diebold and Mariano, 1995). This complements the proper scoring rule framework for evaluating probabilistic forecasts more broadly (Gneiting and Raftery, 2007).

### 8.6 Per-horizon performance breakdown and ablation study

#### 8.6.1 Per-horizon quantitative results

Table 8 reports evaluation metrics disaggregated by prediction horizon (12, 36, and 60 trading days). As expected in financial time-series forecasting, prediction difficulty increases with horizon length, reflected in monotonically rising RMSE and falling R^2^ and MDA values across horizons (Hyndman and Athanasopoulos, 2021; Hochreiter and Schmidhuber, 1997). The 12-trading-day horizon achieves the highest directional accuracy (72%), consistent with the signal-to-noise properties of short-term momentum patterns. The 60-trading-day horizon retains meaningful directional accuracy (62%) above the chance baseline of 50%, confirming that the LSTM signal provides a useful bounded directional tilt even at longer horizons (Heaton et al., 2019). Metrics are computed per horizon independently; ‘Average’ row uses uniform averaging across the three horizon outputs as defined in Section 8.2.

**Table 8:** Per-horizon evaluation metrics on the held-out validation set (27,433 samples).

Horizon	MSE	MAE	RMSE	R^2^	MDA (%)
+12 days	0.0026	0.037	0.051	0.18	72%
+36 days	0.0038	0.043	0.062	0.09	67%
+60 days	0.0051	0.048	0.071	0.04	62%
Average	0.0038	0.043	0.061	0.10	67%

#### 8.6.2 Ablation study

To evaluate the contribution of each major architectural component, we conducted an ablation study by disabling one component at a time and recording the effect on average RMSE and directional accuracy on the validation set. The results are reported in Table 9. Removing the LSTM signal (reverting to a purely historical-drift Monte Carlo) degrades RMSE to 0.073 and MDA to 58%, matching the rolling mean baseline. Removing the bounded signal constraint (allowing the LSTM to freely drive drift) increases RMSE to 0.079 due to overconfident out-of-distribution predictions. These results confirm that both the LSTM signal and the bounding mechanism contribute independently to performance (Heaton et al., 2019; Boyle, 1977). Each row isolates the removal of one architectural element while keeping all others fixed. ‘No health scoring module’ produces identical prediction error but eliminates the retrospective risk-adjusted evaluation layer, confirming that the two pipeline branches are functionally independent as designed.

**Table 9:** Ablation study: component contribution to forecasting performance.

Configuration	Avg RMSE	Avg MDA (%)	Change vs Full
Fullpipeline(LSTM + bounded MC + health module)	0.061	67%	— (baseline)
No LSTM signal (pure historical MC drift)	0.073	58%	+0.012 RMSE/−9% MDA
Unbounded LSTM signal (no tanh/k guardrails)	0.079	63%	+0.018 RMSE/−4% MDA
No health scoring module (prediction-only output)	0.061	67%	No change in RMSE; health metrics unavailable

#### 8.6.3 Extended statistical significance analysis

To strengthen the significance analysis, we applied the Diebold–Mariano (DM) test (Diebold and Mariano, 1995) to compare the proposed LSTM against both baselines using the sequence of out-of-sample squared-error differentials. Results are reported in Table 10. Both comparisons yield statistically significant improvements at the 5% level, confirming that the observed reductions in predictive loss are not attributable to chance variation (Diebold and Mariano, 1995). DM test is applied per the Diebold–Mariano framework (Diebold and Mariano, 1995) using squared-error loss differentials computed on the 27,433-sample held-out validation set.

**Table 10:** Statistical significance tests for forecast comparisons.

Comparison	DM Statistic	*p*-value	Significance
LSTM vs. Persistence baseline	3.42	0.001	*p* < 0.01 (highly significant)
LSTM vs. Rolling Mean baseline	2.15	0.031	*p* < 0.05 (significant)
Paired t-test (LSTM vs. best baseline)	2.89	0.013	*p* < 0.05 (significant)

## 9 Explainable AI integration

### 9.1 Explainable AI integration

Financial forecasting systems are frequently criticized for behaving as “black boxes,” where users can observe outputs but cannot easily verify the underlying reasons (Lipton, 2018; Doshi-Velez and Kim, 2017). For decision support in investing and risk monitoring, interpretability improves trust, supports auditability, and helps identify failure modes (e.g., regime shifts, abnormal volatility, or data gaps). The proposed platform therefore integrates an explainability layer that operates at two levels:

- metric-level transparency, where health score components can be traced to named financial quantities, and
- contextual narrative explainability, where observed historical price changes can be explained by a qualitative analysis of concurrent real-world events - including company performance reports, industry news, macroeconomic changes and market-level events.

The latter layer directly deals with the fundamental interpretability gap in financial AI that serves retailers. Users do not simply require comprehending what a model forecasted, but why a stock moved in a particular manner and what external influences defined that direction (Guidotti et al., 2018; Bussmann, 2020). The subsections below detail the implementation of both layers and qualitatively assess the faithfulness of explanations, consistency and actionability.

### 9.2 Contextual narrative explainability of past price movements

The model-level explainability layer is an implementation of the contextual narrative explanation framework, where the system produces qualitative explanations of observed past price dynamics based on movement grounded on real-world verifiable events. Given an asset, the explainability module is fed with the historical price or NAV series, and the determined health metrics, and produces structured natural-language explanations, which relate price trajectory segments to:

- Company-specific activities: earnings announcements, management transitions, debt restructuring, product announcements, regulatory activity.
- Sectoral developments: industry trends, competitor performance, supply chain disruption, and regulatory changes to the industry.
- Macroeconomic environment: interest rate risk, inflation statistics, exchange rates, central bank policy (especially RBI on Indian assets)
- Trades at the market level: rebalancing of the index, FII/DII flows, market-wide corrections or rallies.

This is based on the contextual explanation paradigm in XAI literature, which acknowledges that numerical attributions are not sufficient to support financial decision making - explanations need to relate model behavior to human-understandable causal explanations to be usable by non-expert users (Guidotti et al., 2018; Bussmann, 2020). Bussmann (2020) confirm that explainability in fintech is both a regulatory and trust factor, rather than a technical one. The contextual narrative layer of this framework is a direct operationalisation of this principle within the context of Indian retail investment.

This approach should not be confused with attribution-based XAI models like SHAP (Mohsin and Nasim, 2025) and LIME (Ribeiro et al., 2016). Attribution techniques provide answers to: “what features of the input did the model attach the largest weight to? - a question useful to model auditing and debugging. Contextual narrative explanation is semantically oriented, as opposed to SHAP or LIME; semantically, contextual narrative explanation can answer the question that a retail investor actually asks, which is: why did this stock perform this way, and what should I look at? (Lipton, 2018; Doshi-Velez and Kim, 2017; Araújo et al., 2025). The second question is more practical with a retail-facing investment analysis tool that uses Indian equity and mutual fund data. The two techniques are complementary; an attribution method might be used as an extrinsic audit tool in later work, and contextual narrative interpretation can be the main end-user interface of interpretability (Lipton, 2018; Guidotti et al., 2018; Bussmann, 2020).

### 9.3 Qualitative assessment of contextual explanations

In order to evaluate the explainability layer, a qualitative assessment was performed on four representative categories of assets based on the Indian equity and mutual funds. Each explanation was assessed based on three criteria in the literature on XAI evaluation (Doshi-Velez and Kim, 2017; Araújo et al., 2025):

- Faithfulness - the explanation accurately relates a verifiable actual event that is concurrent with the actual price movement without creating reasons.
- Relevance - the event mentioned is causally plausible to the type of assets and industry, and not a generic event.
- Actionability - a retail investor can extract a prospective thought on the explanation.
  In all four cases, the generated explanations were based on verifiable market events and never mentioned internal model states or artificial causes. The explanations were not generic but sector specific, thus meeting the relevance requirement. All the explanations included at least one forward-looking signal that a retail investor may apply as a monitoring signal - passing the actionability criterion. To check consistency, the same assets were provided five times with the same inputs. Although phrasing on the surface differed across the runs, the causal event mentioned (e.g., RBI rate cycle, USFDA approvals, tech selloff) was consistent in all five-run cases, and as such, the explanation layer does not hallucinate conflicting causes. Such qualitative assessment of the explainability layer of contextual narrative proves that it is true to the phenomenon of the real-life market dynamics, applicable to the particular asset context, and actionable by the non-expert retail investors (Bussmann, 2020; Bussmann, 2020; Araújo et al., 2025).

### 9.4 Integrated gradients attribution analysis

To provide a quantitative model-level explainability complement to the contextual narrative layer, we implement Integrated Gradients (IG) attribution (Sundararajan et al., 2017) over the 36-step return input window. IG computes the contribution of each input feature (i.e., each lagged daily return in the sequence) to the model’s output prediction by integrating gradients along a path from a baseline input (zero-return sequence) to the actual input. This satisfies the sensitivity and implementation invariance axioms required of a faithful attribution method (Sundararajan et al., 2017).

For a given input sequence x = [r_{t-36}, …, r_{t-1}] and model output F(x) (e.g., the directional signal pred[0]), the Integrated Gradients attribution for the i-th input feature is defined as:

$\begin{array}{l} \mathrm{IG}\_i(x)=(x\_i-x^{’}\_i)\times \int [\alpha =0\;\text{to}\;1]\;\\ (\partial F(x^{’}+\alpha (x-x^{’}))/\partial x\_i)\;\mathrm{d\alpha} \end{array}$

where x’ is the baseline (zero return sequence). In the implementation, the integral is approximated with 50 Riemann steps, which provides stable attribution values consistent with the completeness axiom (sum of attributions equals the output difference from baseline) (Sundararajan et al., 2017).

Table 11 reports the mean absolute attribution weight per input step position, averaged across a representative 500-sample evaluation batch drawn from the validation set. Attribution mass is concentrated in the most recent 8 trading days (steps 29–36), indicating that the LSTM primarily responds to short-term momentum and recent volatility shocks. A secondary attribution cluster appears at steps 18–24 (approximately 3–4 weeks prior), consistent with intermediate mean-reversion patterns documented in the Indian equity literature (Fischer and Krauss, n.d.; Andersen et al., 2003). Values reported as mean ± standard deviation across samples. Higher attribution indicates stronger causal influence on the directional signal output.

**Table 11:** Mean absolute integrated gradients attribution by input window segment, averaged over 500 validation samples.

Input window segment	Step range	Mean |IG| attribution	Interpretation
Recent momentum window	Steps 29–36 (past 8 days)	0.041 ± 0.009	Dominant driver: short-term return momentum and volatility shocks
Intermediate pattern window	Steps 18–24 (3–4 weeks prior)	0.027 ± 0.007	Secondary driver: intermediate mean-reversion and regime signals
Distal history	Steps 1–17 (>4 weeks prior)	0.012 ± 0.005	Low contribution; confirms model does not overfit distant lags

These attribution patterns are consistent across asset classes: equity mutual funds and debt funds show a narrower concentration in the most recent 4–5 steps, reflecting their smoother NAV dynamics, while equities show broader attribution spread across steps 20–36. This asset-class differentiation in attribution behavior provides an additional qualitative validation that the LSTM has learned asset-specific temporal patterns rather than a generic sequence representation (Lipton, 2018; Guidotti et al., 2018; Sundararajan et al., 2017).

The IG visualization is accessible in the web interface as a diagnostic tool for per-asset inspection. Unlike post-hoc SHAP or LIME methods, IG attribution is natively computed at inference time for the same input window used by the Monte Carlo engine, ensuring that the explanation is always consistent with the actual prediction that was made (Ribeiro et al., 2016; Sundararajan et al., 2017; Araújo et al., 2025).

## 10 Discussions of finding

This section describes the outcome of the system and explains why the design decisions are desirable in real-world, risk conscientious forecasting. The discussion centers around four key concepts to include (i) Characteristics of the data and their interpretation, (ii) the behavior of the multi-horizon LSTM outputs, (iii) the significance of Monte Carlo simulation in making predictive decisions under uncertainty and (iv) trust and interpretability.

### 10.1 Implications of dataset coverage and history-length distribution

The statistics of the data sets indicate that the majority of assets are covered nearly on the entire period of time. The group with fewer numbers has fewer histories and days of absence. This is a common inconsistent coverage in market databases, where discrepancies exist in the listing date, business activities, and where data are not collected at all. This has two realistic prediction pipeline limits: (i) the training samples are not uniformly distributed in time across all the assets, and (ii) naive averaging can give a false impression of asset weights when the sampling is not properly controlled (Box and Jenkins, 1976; Tsay, 2010; Bergmeir et al., 2018) The reported coverage and history-length plots are not only helpful in the demonstration of what happened, but also in the reasons why the careful features development and validation is necessary (Diebold and Mariano, 1995; Bergmeir et al., 2018).

The representation in the form of a heatmap that is called a coverage heatmap and usually resembles a collection of tiny vertical gaps is still welcome. Missing periods take the form of narrow discontinuities rather than broad blocks when the assets are very available. This distribution of the accessible lengths of history is a better complementary graphic to read on paper. It presents a clear picture of the number of assets of full-length sequence and shortened sequence.

### 10.2 Multi-horizon prediction behavior and practical interpretation

The LSTM forecaster is trained to forecast the returns will be made at +12, +36 and +60 trading days (Hochreiter and Schmidhuber, 1997; Fischer and Krauss, n.d.). This allows individuals to not over relocate on a single horizon and provides a more relevant forecasting indication as a whole (Heaton et al., 2019). The shorter horizons tend to be with good signal-to-noise ratios compared to the longer horizons whose horizons create more uncertainty and swings with regime changes (Andersen et al., 2003; Fischer and Krauss, n.d.). Consequently, the system does not use the LSTM output as a prediction to a great extent but rather as a direction of one (Heaton et al., 2019; Goodfellow et al., 2016). The decision addresses a well-known issue in financial ML models look good when the data distribution is known, but provide unstable or excessive confidence out of sample predictions when the data distribution is altered (Goodfellow et al., 2016; Heaton et al., 2019; Lipton, 2018).

The deployed pipeline applies explicit clipping, tanh normalization and uses the initial output element as the drift-tilt signal. This helps to restrict the impact of the LSTM even when its predictions are extreme such that the numbers remain constant and do not grow or fall exponentially the following step in the experiment (Boyle, 1977; Glasserman, 2004; Kroese et al., 2011).

### 10.3 Why hybridizing with Monte Carlo improves reliability

Uncertainty cannot be demonstrated by only point predictions that are required in financial decisions. This Monte Carlo forecasting module provides the solution to this by generating a distribution of future frequencies of events (numerous simulated price paths) (Boyle, 1977; Glasserman, 2004; Black and Scholes, 1973; Merton, 1973; Hull, 2018; Glasserman, 2004). This allows you to use percentiles bands and tail-risk statistics (Jorion, 2006; Rockafellar and Uryasev, 2000; Acerbi and Tasche, 2002). The simulator is realistic, because it links the drifting of the baseline and volatility to the history returns and only introduces a certain amount of directional tilt due to MLs, therefore, it is also reflective of the learned model signal (Kroese et al., 2011; Andersen et al., 2003).

This hybrid design has two advantages. First, it enables taking of the simulations of routes to be risk-sensitive in constructs of (volatility, maximum drawdown, and Sharpe-like) ratios, by which portfolio performance is typically gauged (Markowitz, 1952; Sharpe, 1966; Sortino and Price, 1994; Chekhlov et al., 2005). Second, it allows us to easily describe such scenarios such as the median outcome is positive, but tail risk on the downside remains significant, and this cannot be described as only having one predicted value (Rockafellar and Uryasev, 2000; Acerbi and Tasche, 2002)

### 10.4 Interpretability and trust: linking outputs to financial concepts

The interpretability layer is stronger because the main outputs are based on common, named metrics, including return, volatility, Sharpe, Sortino, and drawdown (Sharpe, 1966; Sortino and Price, 1994). Consequently, checking a health score and risk summaries becomes much simpler for consumers, which effectively dismantles the “black box” barrier (Lipton, 2018; Guidotti et al., 2018). Local attribution visualization (Integrated Gradients over the 36-step window) additionally delivers model-level diagnostic that indicates whether forecasts are made on the basis of very recent return shocks or on wider short-term trends (Sundararajan et al., 2017). In finance, such diagnostics are very important because model behavior can vary greatly with the volatility regime. The most significant design principle is probably that any explanation in natural language should be faithful to the signals calculated and metrics reported. This means that unverifiable event-based statements (e.g., “the move was caused by earnings news”) should not be used unless the external event dataset is very clearly included. This limitation makes it straightforward to verify the reports and minimizes the chances of providing wrong information in user reports (Guidotti et al., 2018; Bussmann, 2020).

### 10.5 Constraints and threats to validity

There are several limitations that should be mentioned. A simple holdout is also the method of splitting training; it is efficient, but time-series forecasting should be evaluated using walk-forward (rolling-origin) validation to represent the reality of actual deployment (Bergmeir et al., 2018). Secondly, the prediction job uses univariate return sequences. Adding more covariates, such as volume, market index returns, and macro indicators, could make it work better, but it would also make it more complicated and need more data. Third, the Monte Carlo part assumes that short-term return statistics stay roughly the same within each simulation segment and employs constrained controls to deal with regime extremes (Boyle, 1977; Glasserman, 2004). In times of crisis, more advanced regime-switching or volatility models may be needed to make the simulation more realistic (Engle, 1982; Bollerslev, 1986). Even with these problems, the system architecture is still good for a prototype that focuses on publishing because it puts reproducibility, stability, interpretability, and a clear distinction between learnt signal extraction and uncertainty-aware forecasting first (Heaton et al., 2019; Guidotti et al., 2018).

## 11 Conclusion

In this paper we have given a framework that can be constructed easily and utilized in the check of health of stocks and the prediction of returns on the same. It unites four components: (i) one method that evaluates financial data using simple metrics (Markowitz, 1952; Sharpe, 1966), (ii) a method that, using an LSTM neural network, draws out signals of a sequence of time horizons into the future (Hochreiter and Schmidhuber, 1997), (iii) a Monte Carlo simulation that predicts various possible future scenarios (Boyle, 1977; Glasserman, 2004), and (iv) a layer that documents the results enough to be audited by the process (Guidotti et al., 2018; Bussmann, 2020). The entire system receives historical price and NAV information, converts it into standardized return windows, transforms the multi-horizon forecasts and finally transforms the multi-horizon forecasts into health and risk summaries that are easy to read and display in a web page.

The practical implications of this research framework deserve a detailed discussion to improve the practical aspects of the paper. It is suggested to make the conclusion longer by referring to the following stakeholder groups specifically:

- Retail Investors: what can individual investors do with the health score and probabilistic scenario distributions coming from the Monte Carlo simulation to make better buy, hold, or exit decisions? A quick example showing a retail investor interpreting a health score along with the CAGR forecast range would be very helpful.
- Financial Analysts: how can the explainability layer—linking LSTM attributions to macroeconomic forces and sectoral developments—be used in analysts’ due diligence processes or investment research reports?
- Portfolio Managers: in what respect can the framework’s probabilistic outputs (Sharpe/Sortino distributions, drawdown risk estimates) be helpful for risk budgeting, asset allocation optimization, and portfolio rebalancing decisions. Besides, practical deployment constraints should also be included in the discussion, such as data latency requirements, model retraining frequency, and the minimum historical data length necessary for reliable scoring and forecasting of different asset classes.
  The forecasting component is configured as a monitored learning problem having numerous outputs (Hochreiter and Schmidhuber, 1997). It takes as input a series of 36 past daily returns, and makes predictions at the 12, 36 and 60 trading day horizons (Fischer and Krauss, n.d.). It is fed through a small LSTM model which is subsequently incorporated into a forward-simulation module. Rather than considering the neural output as a free-form drift, it is treated only as a small directional signal that modulates the already obtained drift (Heaton et al., 2019). This makes the forecasts predictable and does not have impractical long-run projections. It employs Monte Carlo simulation to sample scenario distributions and calculate risk measures like CAGR, volatility, Sharpe ratio and Sortino ratio and maximum drawdown (Sharpe, 1966; Sortino and Price, 1994; Chekhlov et al., 2005; Boyle, 1977). It is well-known that the metrics can be handy in coping with the uncertainty that is inherent in financial forecasting. Scenario data and metrics information are blended to develop a composite Health Score, which depicts the past performance of assets and indicates the potential of their future performance. Finally, the study adds an explainability component that fosters transparency by (i) disclosing the financial metrics used in the score (Sharpe, 1966; Sortino and Price, 1994), (ii) disclosing the scenario uncertainty in percentile ranges, and (iii) giving a visualization of model attribution through the 36-step input window for debugging and getting a more insightful explanation (Sundararajan et al., 2017).

## Data Availability

The original contributions presented in the study are included in the article/Supplementary material, further inquiries can be directed to the corresponding author/s.
